# Mycotoxins, gut microbiota alterations and liver disease in animals: A scoping review

**DOI:** 10.1007/s10565-026-10156-5

**Published:** 2026-02-09

**Authors:** Álvaro Lázaro, Massimo Frangiamone, Marcelo de las Heras, María José Ruiz

**Affiliations:** 1 Laboratory of Food Chemistry and Toxicology, Faculty of Pharmacy and Food Science, University of Valencia, Av. Vicent Andrés Estellés S/N, 46100 Burjassot, València, Spain; 2 Department of Biomedical Sciences, University of Lausanne, Rue du Bugnon 7, 1005 Lausanne, Switzerland; 3 Department of Animal Pathology, Faculty of Veterinary, University of Zaragoza, C. de Miguel Servet, 177, 50013 Saragossa, Spain; 4 Research Group in Alternative Methods for Determining Toxic Effects and Risk Assessment of Contaminants and Mixtures (Risktox), Faculty of Pharmacy and Food Science, University of Valencia, Av. Vicent Andrés Estellés S/N, 46100 Burjassot, València, Spain

**Keywords:** Mycotoxins, Microbiota, Probiotics, Hepatotoxicity and animal pathology

## Abstract

Mycotoxins are fungal secondary metabolites widely detected in up to eighty percent of frequently consumed foods, strongly associated with toxicological mechanisms. Evidence indicates that hepatic pathophysiology entails gut microbiota dysbiosis mediated by the complex, bidirectional interactions within the gut–liver axis. This scoping review aims to provide insight into the relationship between mycotoxins, gut microbiota, and liver disease in animals, having been conducted following the Preferred Reporting Items for Systematic Reviews and Meta-Analyses (PRISMA) guidelines (n = 44). The analyzed species were hens, broilers, rabbits, mice, carps, turbots, *Lateolabrax maculatus*, chicks, sheep, and rats. The most altered liver parameters, as a consequence of mycotoxin exposure, were alkaline phosphatase, alanine aminotransferase, aspartate aminotransferase, malondialdehyde, reactive oxygen species, superoxide dismutase, glutathione peroxidase, tumor necrosis factor-α, lipopolysaccharide, and inflammatory infiltration. Gut microbiota changes were analyzed at phylum (Firmicutes, Bacteroidetes, Proteobacteria, Actinobacteria and Verrucomicrobia) and genus level (*Bifidobacterium, Lactobacillus, Clostridium, Ruminococcus, Akkermansia, Escherichia, Allobaculum, Blautia, Staphylococcus, Prevotella, Bacteroides, Turicibacter, Corynebacterium, Roseburia, Coprococcus*). What is more, out of more of 400 existing mycotoxins, only a small fraction of mycotoxins has been investigated in the interplay of the gut-liver axis ((Aflatoxin B1 (AFB1), ochratoxin A (OTA), deoxynivalenol (DON), zearalenone (ZEN), enniatins (ENNs) and T-2 toxin)). Therefore, more research is to better understand the interplay of interactions regarding mycotoxins and the gut microbiota-liver axis, focusing on the formulation of new functional foods and/or nutraceuticals as toxicity mitigating strategies.

**Graphical Abstract:**

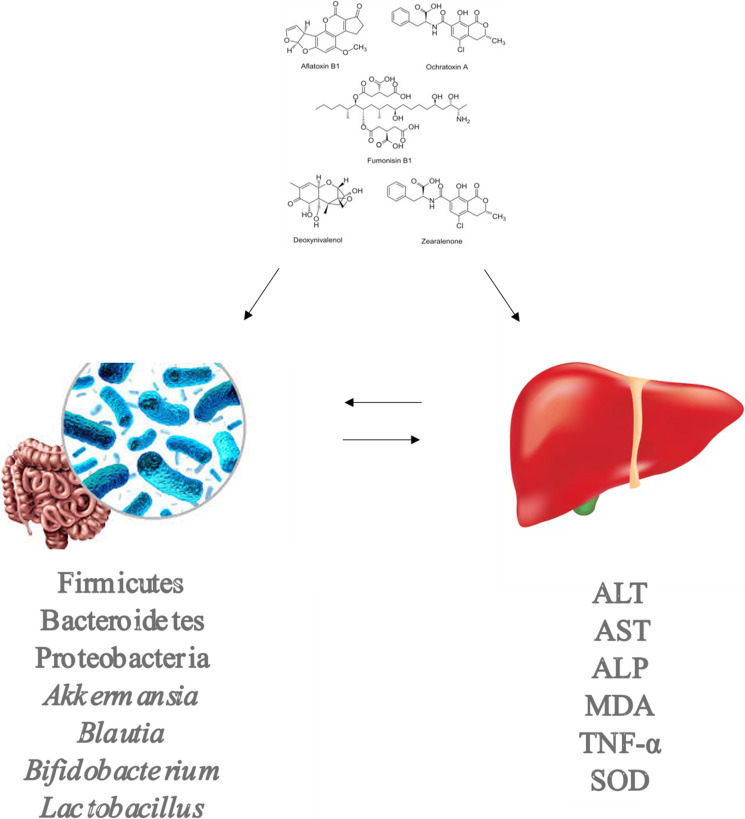

**Supplementary Information:**

The online version contains supplementary material available at 10.1007/s10565-026-10156-5.

## Introduction

Mycotoxins are toxic secondary metabolites produced by filamentous fungi and are estimated to contaminate up to 80% of global diets (Eskola et al. 2020). Although they are synthesized by a wide range of fungal species, the genera *Aspergillus*, *Fusarium*, and *Penicillium* are recognized as the principal producers. Exposure to mycotoxins poses significant risks to both human and animal health, being associated with neurotoxicity, hepatotoxicity, nephrotoxicity, teratogenicity, carcinogenicity, reproductive disorders, and immunosuppression. As dietary intake represents the primary route of human exposure, continuous monitoring of mycotoxin levels in food and feed is essential to ensure food safety (Awuchi et al. 2022; Frangiamone et al. 2023; Lázaro et al. 2024a, b; Pleadin et al. 2019; Saleemi et al. 2023).

To mitigate these risks, the European Union has established maximum permissible levels for several mycotoxins in food through Commission Regulation (EU) 2023/915 of 25 April 2023. This regulation covers aflatoxins (AFB1, AFB2, AFG1, AFG2, and AFM1), citrinin (CIT), deoxynivalenol (DON), ergot sclerotia and ergot alkaloids, fumonisins (FB1 and FB2), HT-2 and T-2 toxins, ochratoxin A (OTA), patulin, and zearalenone (ZEN) (European Commission 2023). In contrast, a growing group of compounds referred to as emerging mycotoxins remains largely unregulated (Lázaro et al. 2024b). This group includes altertoxin, altuene, alternariol (AOH), alternariol monomethyl ether (AME), aurofusarin, beauvericin (BEA), diacetoxyscirpenol, enniatins (ENNA, ENNA1, ENNB, ENNB1), fusaproliferin, fusarenon X, fusaric acid, moniliformin, neosolaniol, phomopsin, rugulosin, sterigmatocystin (STG), tenuazonic acid (TeA), and tentoxin. Notably, guidance values for selected *Alternaria* toxins (AOH, AME, and TeA) in specific food matrices were introduced through Commission Recommendation (EU) 2022/553 (European Commission 2022), and new maximum levels for T-2 and HT-2 toxins in oats have recently been established (European Commission 2024).

**Fig. 1:**
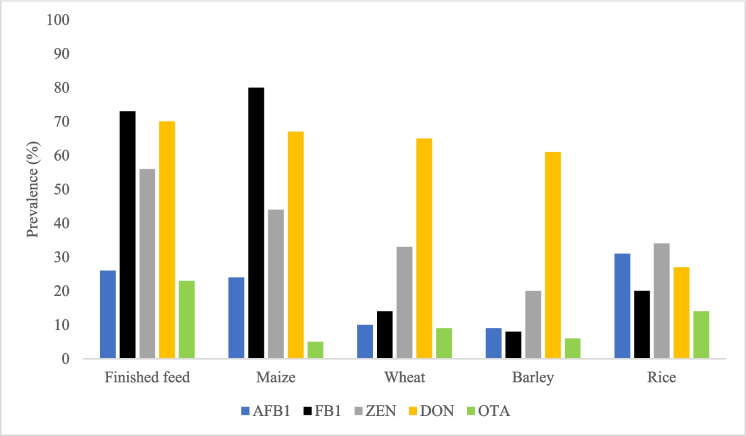
Global mycotoxin occurrence in different commodities. The figure shows that all major mycotoxins are widely detected across diverse food and feed commodities, with notable variability in prevalence among regions and toxin types. Data was obtained from Kovač et al. (2022); Tian et al. (2022); Tolosa et al. (2021) and Vila-López et al. (2023). AFB1:aflatoxin B1; DON:deoxynivalenol; FB1:fumonisin B1); OTA:ochratoxin A; ZEN:zearalenone. Blue for AFB1, black for FB1, grey for ZEN, yellow for DON, and green for OTA

Despite regulatory efforts, mycotoxins remain widely distributed across food and feed commodities worldwide. As illustrated in Fig. 1, contamination has been reported in many of the most commonly consumed products by humans and livestock (Tian et al. 2022). Among animal feedstuffs, AFB1, FB1, ZEN, DON, and OTA are the most prevalent mycotoxins and are frequently detected at concentrations exceeding established regulatory limits (Santos Pereira et al. 2019). AFB1, in particular, is the mycotoxin most commonly reported above EU maximum levels, while ZEN, T-2, and HT-2 toxins have also been shown to exceed permitted concentrations in several surveys (Santos Pereira et al. 2019).

The widespread occurrence of mycotoxins presents a major challenge to food safety, prompting the development of various physical, chemical, and biological decontamination strategies (Tian et al. 2022). Conventional chemical and physical treatments can reduce mycotoxin levels but may leave undesirable residues or compromise food quality. Thermal processing is often ineffective due to the heat stability of many mycotoxins (Murtaza et al. 2024a, b). Consequently, emerging technologies such as cold plasma treatment have gained increasing attention. Cold plasma generates reactive chemical species capable of rapidly inactivating microorganisms at ambient temperatures without producing harmful residues, thereby improving food safety and extending shelf life. The integration of such innovative approaches with conventional methods may enhance mycotoxin mitigation while preserving nutritional and sensory quality (Murtaza et al. 2024a, b; Murtaza et al. 2024b, 2025).

Beyond their direct toxic effects, mycotoxins are now recognized as important modulators of the gut microbiota, a complex and dynamic microbial community essential for host health (Thursby & Juge 2017). The gut microbiota is increasingly considered an “emerging organ,” shaped by factors such as diet, stress, physical activity, medication use, and exposure to environmental contaminants, including mycotoxins (Donati Zeppa et al. 2019; Góralczyk-Bińkowska et al. 2022; Mousa et al. 2023; Wilson et al. 2020; Xia et al. 2022). Its relevance is underscored by its vast genetic potential—exceeding four million microbial genes compared with approximately 26,600 human genes—and by the exceptionally high microbial density in the colon, the highest of any known ecosystem on Earth (Hu et al. 2016; Rinninella et al. 2019).

Disruptions in gut microbiota composition and function have been linked to a wide range of diseases, including inflammatory bowel disease, neurodegenerative disorders, metabolic diseases, mental health disorders, and cancer (An et al., 2023; Anand et al., 2022; Ancona et al., 2021; Camilleri, 2019; Daily et al., 2021; Lang et al., 2020; Menozzi et al., 2021; Micic et al., 2023). These effects are mediated through bidirectional gut microbiota–host communication networks, known as microbiota-related axes, such as the gut–brain, gut–immune system, and gut–liver axes (Moughnyeh et al., 2021; Nalage et al., 2023; Stojanov et al., 2020; Yang et al. 2018). Among these, the gut microbiota–liver axis is particularly relevant in the context of mycotoxin exposure, as the liver plays a central role in metabolism, immune regulation, and detoxification of xenobiotics, including dietary toxins (Trefts et al. 2017).

The gut–liver axis is anatomically sustained by the portal vein, which transports gut-derived microbial products directly to the liver, and functionally regulated by hepatic feedback mechanisms such as bile acid secretion and immune signaling (Albillos et al. 2020). Environmental stressors—including dietary patterns, alcohol consumption, and toxic exposures—can disrupt gut microbial homeostasis, impair intestinal barrier integrity, and increase intestinal permeability. This allows microbial components and metabolites to translocate to the liver, where they activate inflammatory and metabolic pathways that contribute to liver injury and disease progression (Hsu & Schnabl 2023; Lázaro et al., 2025; Li et al., 2024; Manothiya et al. 2025; Mohr et al. 2022).

In this context, increasing evidence suggests that mycotoxins can simultaneously alter gut microbiota composition and exacerbate liver toxicity, thereby acting through the gut microbiota–liver axis (Aytekin Sahin et al. 2022; Bai et al. 2021; Cao et al. 2022; Chang et al. 2020; Chlebicz & Śliżewska 2020; Deepthi et al. 2017; Du et al. 2021; Fang et al. 2020; Izco et al. 2021; Jia et al. 2021; Khalafalla et al. 2022; Kumara et al. 2020; Li et al. 2022; Liao et al. 2018; Markoviaw et al., 2019; Maranghi et al., 2018; Mateos et al., 2018; Murtaza et al. 2023a, b; Murtaza et al. 2023b; Novak et al. 2021; Nowak et al. 2025; Poloni et al. 2021; Rushing & Selim 2019; Saad-Hussein et al. 2021; Suo et al. 2023; Vornoli et al. 2022; Wang et al. 2016; Wang et al. 2019a, b; Wang et al. 2022a, b; Wang et al. 2024a, b; Yan et al. 2022; Yasmeen et al. 2021; Ye et al. 2024; Yu et al. 2022; Zhai et al. 2021; Zhan et al., 2022; Zhang et al. 2020; Zhang et al. 2021; Zhang et al. 2022a, b; Zheng et al. 2021). While numerous studies have independently examined mycotoxin-induced dysbiosis or hepatotoxicity, integrated analyses of their combined effects on the gut–liver axis remain limited and fragmented (Heikkinen et al. 2025; Huybrechts et al. 2024). Differences in experimental models, exposure conditions, and analytical approaches further complicate interpretation. Moreover, interactions between many regulated and emerging mycotoxins and the gut microbiota–liver axis have yet to be systematically explored (Guerre 2020; Liew & Mohd-Redzwan 2018; Zhai 2020; Zhai 2025).

Therefore, the aim of this scoping review is to comprehensively examine the impact of mycotoxin exposure on the gut microbiota–liver axis in animal models. By synthesizing current evidence on how mycotoxins concurrently influence gut microbial communities and hepatic function, this review seeks to clarify underlying mechanisms that can help the development of innovative functional foods or nutraceutical strategies to mitigate mycotoxin-induced toxicity.

## Material and method

### Search strategy

In order to reduce the possible bias, in this scoping review was performed the Preferred Reporting Items for Systematic Reviews and Meta-Analyses (PRISMA) statement protocol for screening titles, abstracts, and full texts (Page et al. 2021). A scoping review of the literature was conducted in March–April 2025, and the last search was completed on 30 April 2025. Using three databases (PubMed, Web of Science, and Scopus) without time restriction to provide the whole mycotoxins state of the art in the framework of gut microbiota and liver disease in animals.

For each database, the search was executed using the “All Fields” option (or the equivalent setting, ensuring that keywords were searched across titles, abstracts, keywords, and any available indexed fields). Discrepancies between reviewers were resolved by consensus, with a third reviewer consulted when necessary. Quality assessment was focused on study design, experimental protocols, outcomes measurement and reporting quality of the article.

The scoping review was conducted using the following search strings:

1. Refined research of PubMed, Web of Science, and Scopus. Period: All years.

(´´Aflatoxin B1´´ OR ´´Aflatoxin B2´´ OR ´´Aflatoxin G1´´ OR ´´Aflatoxin G2´´ OR ´´Aflatoxin M1´´ OR ´´Ochratoxin A´´ OR ´´Deoxynivalenol´´ OR ´´Zearalenone´´ OR ´´Patulin´´ OR ´´Fumonisin B1´´ OR ´´Fumonisin B2´´ OR Enniatins´´ OR ´´Enniatin A´´ OR ´´Enniatin B´´ OR ´´Enniatin A1´´ OR ´´Beauvericin´´ OR ´´Fusaric acid´´ OR ´´Moniliformin´´ OR ´´Fusarenon X´´ OR ´´Aurofusarin´´ OR ´´Fusaproliferin´´ OR ´´Diacetoxyscirpenol´´ OR ´´Alternariol monomethyl ether´´ OR ´´Tenuazonic acid´´ OR ´´Altertoxin´´ OR ´´Tentoxin´´ OR ´´Citrinin´´ OR ´´Sterigmatocystin´´ OR ´´Neosolaniol´´ OR ´´Citreoviridin´´ OR ´´Rugulosin´´ OR ´´Phomopsin´´) AND (´´liver´´) AND (´´microbiota´´).

The selection of non-regulated mycotoxins considered in this study was based on the criteria proposed by Mihalache et al. (2023) and on reports issued by the Spanish Agency for Food Safety and Nutrition (AESAN) (AESAN, 2025). It is acknowledged that additional non-regulated fungal metabolites exist that were not included in the present analysis. Nevertheless, it was assumed that the spectrum of compounds evaluated was sufficiently broad to encompass the main non-regulated mycotoxins of relevance.

All data summarized in tables and figures were extracted from the publicly available studies included in this review (n = 44). Quality control and verification were ensured by cross-checking all extracted information against the original publications. Full references for each data source are provided in the reference list and figure legends.

### Scoping review process

Among the 283 reports found, 123 were retrieved from PubMed, 82 from Web of Science, and 78 from Scopus. A total of 99 duplicate records were identified and removed. In addition, during the screening of titles and abstracts, another 31 records were excluded from the scoping review. 153 reports were assessed for eligibility.

**Fig. 2:**
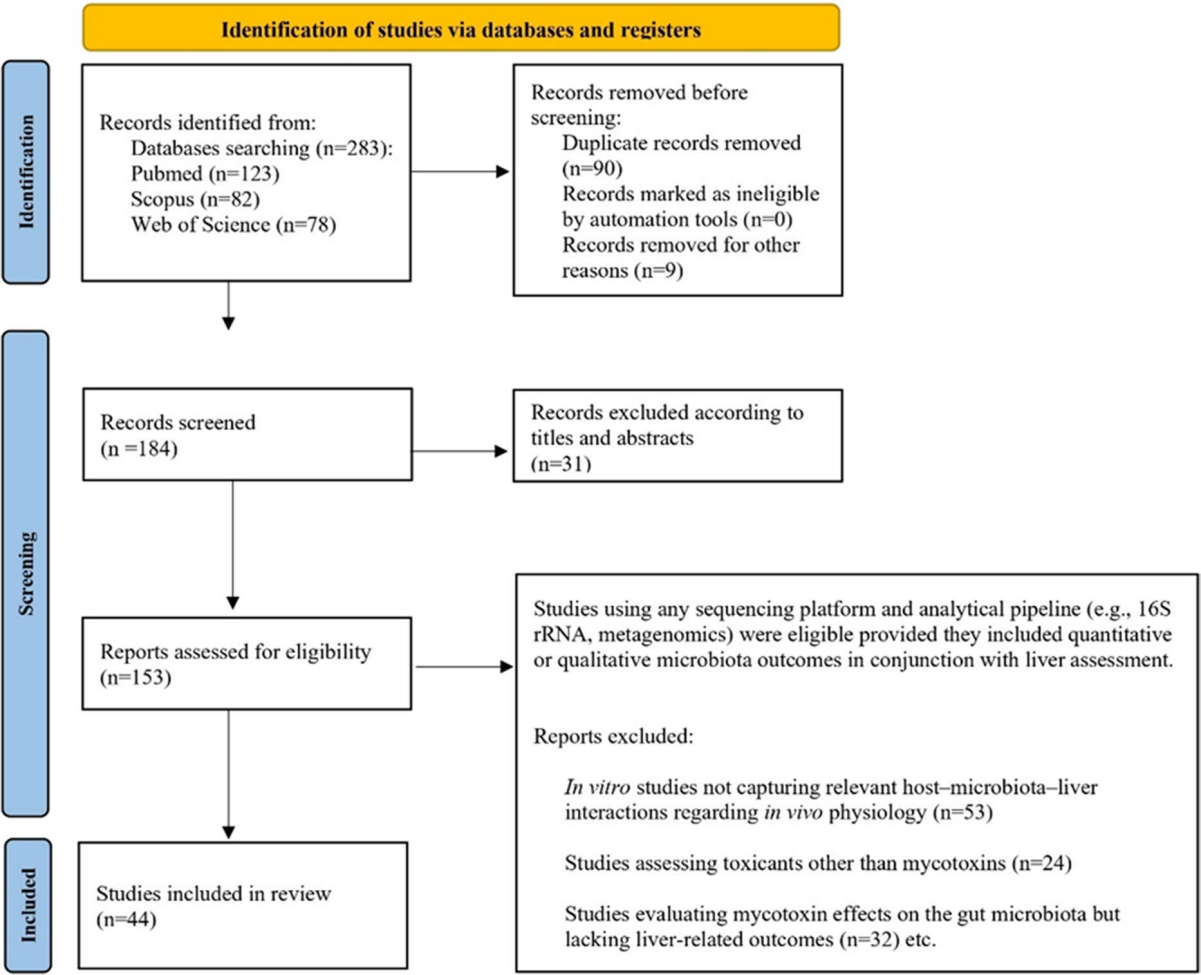
PRISMA flow diagram summarizing the literature screening process. The diagram illustrates the total number of articles identified, screened, and included across the two bibliographic search strategies, based on the keyword combinations and year ranges described in the Methods. Final sample sizes are indicated (n)

The scoping review included in vivo animal studies that simultaneously evaluated the effects of mycotoxin exposure on the gut microbiota and liver-related outcomes. Eligible studies had to report both microbiome measures (e.g., taxonomic composition) and hepatic parameters (e.g., histopathology, enzyme activity, oxidative stress markers). No restrictions were applied regarding animal species, mycotoxin type, dose, or duration of exposure. Studies using any sequencing platform and analytical pipeline (e.g., 16S rRNA, metagenomics) were eligible provided they included quantitative or qualitative microbiota outcomes in conjunction with liver assessment. In vitro experiments were removed because they do not capture host–microbiota–liver interactions relevant to in vivo physiology (53 studies excluded). Studies investigating toxicants other than mycotoxins were also excluded, as they did not meet the exposure criteria of this review (24 studies excluded). Also, studies evaluating mycotoxins and gut microbiota but lacking liver-related outcomes were excluded because they did not address the dual-focus research question (32 studies excluded). Finally, 44 studies (ranging from 2018 to 2025) were considered appropriate for inclusion in the present scoping review, assessed and classified based on all the types of mycotoxins found: AFB1, OTA, DON, ZEN, ENNs, BEA and T-2 (Fig. 2).

### Data conversion

To ensure comparability across studies, all exposure metrics were converted to feed-based concentrations and expressed as either µg/kg feed or mg/kg feed. When doses were originally reported on a body-weight basis, they were converted to feed concentrations using body weight and feed intake information provided in the original studies or, when unavailable, default species-specific values.

Daily intake of the compound was estimated as:

$$\text{Daily intake}={\mathrm{Dose}}_{\mathrm{bw}}\times \text{Body weight}$$

where Dose _bw_ is expressed as µg/kg body weight per day and body weight is expressed in kilograms.

The corresponding concentration in feed was calculated as:

$${\mathrm{Dose}}_{\mathrm{feed}}=\text{Daily intake}/\text{ Feed intake}$$

where feed intake is expressed in kilograms of feed consumed per day, resulting in a concentration expressed as µg/kg feed.

When necessary, feed concentrations were converted to mg/kg feed by dividing by 1,000. These steps can be combined into a single expression:

$$Dose_{feed}\left(mg/kg\;feed\right)=\left(Dose_{bw}\left(\mu g/kg\;bw/day\right)\times Body\;weight\right)/\left(Feed\times1,0000\right)$$

### Limitations and Potential Publication Bias

We acknowledge several limitations in this scoping review. First, a quantitative synthesis of microbiota changes (e.g., exact increases or decreases in bacterial taxa) was not possible, as some studies reported only qualitative data. Additionally, variability in animal species, exposure times, doses, and experimental designs may have influenced the observed outcomes. Finally, as with any scoping review, potential publication bias cannot be excluded, since studies with significant or positive results are more likely to be published than those with null findings.

## Results and discussion

### Mycotoxins, Gut Microbiota, and Liver

#### AFB1

AFB1 exposure consistently induced hepatic oxidative stress and functional impairment across multiple species, including poultry (hens, broilers), rabbits, mice, and fish (Cao et al. 2021; Huang et al. 2023; Jia et al. 2024; Lin et al. 2023; Liu et al. 2022, 2023; Qing et al. 2022; Sui et al. 2022; Tao et al. 2023; Xu et al. 2023a, b; Xue et al. 2023; Ye et al. 2024; Zhang et al. 2023).

##### Hepatic injury and oxidative stress

Liver enzymes such as ALT, AST, and ALP were significantly elevated in a dose-dependent manner, reflecting hepatocellular injury. Lipid peroxidation markers, MDA and ROS were increased, while antioxidant defenses, including SOD and GSH-Px, were decreased. Histopathological evaluation revealed hepatocyte hypertrophy, vacuolar and fatty degeneration, necrosis, and inflammatory cell infiltration, consistent with oxidative stress-induced cytotoxicity.

##### Gut microbiota dysbiosis induced by AFB1

In parallel with hepatic injury, AFB1 altered substantially gut microbiota. At the phylum level, Proteobacteria and Actinobacteria increased, Bacteroidetes decreased, and Firmicutes and Verrucomicrobia exhibited variable changes (Cao et al. 2021; Guo et al. 2023; Huang et al. 2023; Jia et al. 2024; Karimi Torshizi & Sedaghat 2023; Kasmani et al. 2023; Lin et al. 2023; Liu et al. 2022; Liu et al. 2023; Peng et al. 2022; Peng et al. 2025; Qing et al. 2022; Subramaniam et al., 2018; Sui et al. 2022; Tao et al. 2023; Xu et al. 2023a, b; Xue et al. 2023; Ye et al. 2024; Zhang et al. 2023). At the genus level, *Bifidobacterium, Lactobacillus, Clostridium*, and *Escherichia* increased, whereas *Prevotella, Akkermansia,* and *Roseburia* decreased, indicating a shift toward pro-inflammatory and dysbiotic profiles (Tamanai-Shacoori et al. 2017).

##### Dose–effect analysis

Across studies, no clear dose-dependent patterns were observed across increasing AFB1 concentrations, likely due to variability in animal species, experimental conditions, and exposure durations. In contrast, hepatic outcomes showed a consistent trend: all tested AFB1 doses induced oxidative stress and contributed to liver damage. Adding to the complexity, contradictory results have been reported for OTA and AFB1 in Caco-2 cells. This discrepancy may be explained by the antagonistic effects observed when Caco-2 cells were exposed to AFB1 in combination with OTA (Sobral et al. 2018).

##### Mechanistic involvement of the gut–liver axis

Mechanistically, increased Proteobacteria likely enhances LPS translocation, triggering TLR4-mediated inflammatory signaling, which synergizes with oxidative stress to damage hepatocytes (Cordiano et al., 2023; Mohammad et al., 2020; Rizzatti et al. 2017; Vasques-Monteiro et al. 2021; Vester-Andersen et al. 2019; Shen et al. 2021). Decreased Bacteroidetes and SCFA-producing bacteria compromise intestinal barrier function and energy metabolism, exacerbating hepatotoxicity (Wu et al. 2025). Interestingly, increases in *Lactobacillus* and *Bifidobacterium* may represent compensatory responses to AFB1-induced gut perturbations, aiming to limit toxin absorption and inflammation (Culp & Goodman 2023; Choi et al. 2025).

##### Adaptive and compensatory microbial responses

Interestingly, AFB1 exposure has been reported to increase the abundance of health-promoting bacteria such as *Lactobacillus* and *Bifidobacterium* (Dempsey et al., 2022). Notably, *L. plantarum* has demonstrated a high capacity to detoxify AFB1 in vitro (up to 89.5%), whereas several *Bifidobacterium* strains (*Bf. bifidum* BGN4, *Bf.* sp. JO3, *Bf. longum* JR20, *Bf.* sp. CH4, *Bf. adolescentis* 14, and *Bf.* sp. Bf6) have shown detoxification efficiencies ranging from 26 to 45% in vitro. However, the well-recognized discrepancies between *in vitro* and *in vivo* studies remain a major limitation when comparing results across studies. Although AFB1 is not an antibiotic, it may exert selective antimicrobial effects on certain bacterial taxa, indirectly favoring the proliferation of more tolerant genera such as *Lactobacillus* and *Bifidobacterium* (Choi et al. 2025). Additionally, microbial interactions within the gut ecosystem may further contribute to these divergent findings. For instance, *Streptococcus* species produce streptolysin S, a toxin capable of degrading lipoteichoic acid and exerting cytotoxic effects on specific *Lactobacillus* species (Dai et al. 2023). Collectively, these selective antimicrobial effects and interbacterial interactions may help explain the contradictory results reported for *Lactobacillus* abundance following AFB1 exposure.

##### Modulation of the gut–liver axis as a therapeutic strategy

Interventions with antioxidants such as vitamin E or *Moringa oleifera* attenuated oxidative stress and histological damage, confirming the central role of ROS in AFB1 hepatotoxicity (Saleemi et al. 2023). Similarly, postbiotics and probiotic strains (YCWE, *Lactobacillus,* *Bifidobacterium*) reduced AFB1 bioavailability and restored gut microbiota balance, mitigating liver injury (Wang et al. 2025; Yu et al. 2025). All information is collected in Table 1, Table 1S, Figs. 1S, 2S and 3S.

**Table 1:** Overview of key characteristics of studies investigating the effects of AFB1 on the interplay between microbiota and liver disease. The table summarizes animal models, administered doses, exposure durations, experimental approaches, and main findings reported across studies, highlighting the heterogeneous methodologies and outcomes in the current literature

Animal	Dose administration	Exposure time	Sample location	Experimental assay	Results		References
					**Microbiota**	**Liver**	
**Birds**							
768 Arbor Acres broilers, 14 days old	69 µg/kg feed	3 weeks (Sub-chronic)	Jejunum	Serum biochemical parameters, antioxidant indices in serum and liver and microbiota analysis through V3 and V4 hypervariable regions	At the phylum level, AFB1 increased Proteobacteria*,* but reduced Firmicutes, Bacteroidetes and Actinobacteria. Also, AFB1 exposure significantly reduced the relative abundance of *Lactobacillus*, while that of *Escherichia* was significantly increased compared to control	AFB1 led to an increase in AST and ALT. However, AFB1 exposure decreased SOD and GSH-Px in contrast to control	Jia et al. 2024
144 one-day-old male broilers	500 µg/kg feed	3 weeks (Sub-chronic)	Ileum	Serum biochemistry, histology, and enzyme activity analysis and gut microbiota analysis through V3 and V4 hypervariable regions	At the genus level, AFB1 augmented *Bacteroides*, *Lactobacillus* and *Enterococcus*, but diminished *Clostridium* in comparison to control	AFB1 enhanced ALT, but reduced AST and ALP comparing with control	Peng et al. 2025
164 one-day-old broilers	3000 µg/kg feed	4 weeks (Sub-chronic)	Cecum	Histopathological and intestinal lesion scoring, serum biochemistry analyses, and 16S rRNA sequencing through V3 and V4 hypervariable regions	At the phylum level, AFB1 enhanced Bacteroidetes*,* Actinobacteria, and Proteobacteria and reduced Firmicutes. AFB1 increased the abundance of *Bacteroides, Rikenellaceae,* but decreased the abundance of *Alistipes*, *Lactobacillus*, *Ruminococcus,* and *Eubacterium* regarding the control	AFB1 affected the liver including intrahepatic hemorrhage, inflammatory cell infiltration, and fatty degeneration compared with the control group. Meanwhile, AFB1 administration increased the content of MDA and decreased the content of GSH, GSH-Px, and SOD compared with control	Tao et al. 2023
200 one-day-old Ross 308 broiler chicks	2,500 µg/kg feed	6 weeks (Sub-chronic)	Ileum	Serum enzyme activity, liver oxidative stress and antioxidant parameters and microbial population	Total bacterial population and lactic acid bacteria in ileum diminished in AF group, while the highest coliform bacterial population was observed in this group	The use of AFB1 in diet consistently elevated the levels of LDH, GGT, AST, ALT, ALP, MDA, and NO in blood, but decreased SOD, CAT and GPx regarding the control	Kasmani et al. 2023
Ninety male Arbor Acres broiler chickens	1,250 µg/kg feed	1 week (Sub-chronic)	Duodenum	Biochemical analysis, determination of related gene expression levels, and sequencing of the 16S rRNA gene and microbiota data analysis	Compared with the control, AFB1 showed a significant increase in the relative abundance of Bacteroidetes, Proteobacteria, and Verrucomicrobia, and a markedly decrease of the relative abundance of *Tenericutes*. At the genus level, *Lactobacillus*, *Bacteroides*, *Blautia*, *Akkermansia,* and *Ruminococcus* were drastically increased in the AFB1 exposure, but notably decreased the relative abundance of *Faecalibacterium* and *Subdoligranulum*	ALT, AST, and MDA were higher in the AFB1 exposure, while the activities of the antioxidant enzymes SOD and GPX1 were reduced compared with control	Huang et al. 2023
180 one-day-old Jingfen No. 1 commercial chicks	20 μg/kg feed	6 weeks (Sub-chronic)	Cecum	Analysis of biochemical parameters in serum, kidney and liver, and 16S rRNA gene sequencing of the gut microbiota	At the phylum level, AFB1 caused a Bacteroidetes increase and a Firmicutes decrease. At a genus level, AFB1 decreased *Lactobacillus* and *Bacteroides* and increased *Ruminococcus* and *Blautia* in contrast to the control	As for liver damage parameters, AFB1 enhanced ALP and ASP, but reduced ALT. Also, AFB1 increased ROS, GSH, GSSG, IL-6, IL-8, and TNF-α in a significant manner, but decreased IL-10 and SOD compared with the control. On the other hand, GSH-Px values were similar in both AFB1 exposure and control	Qing et al. 2022
84 32-wk-old Bovans white laying hens	500 μg/kg feed	12 weeks (Sub-chronic)	Ileum	Bacteria and liver fat content determination	The greatest population of total aerobic (8.97 log10 CFU/g) and lactic acid bacteria (8.30 log10 CFU/g) was observed in the control, while AFB1 showed a slight reduction in both of them, (8.63 log10 CFU/g and (8.26 log10 CFU/g, respectively)	There was a significant increase in the liver fat content in AFB1 exposure (42.21%) compared to the control one (30.64%)	Karimi Torshizi & Sedaghat 2023
300 one-day-old Ross broilers	4–42 μg/kg feed	3 weeks (Sub-chronic)	Feces	Determination of antioxidant indexes in serum, organ index, HE staining and intestinal analysis by 16S rRNA through V3 and V4 hypervariable regions (SILVA database, sequence depth to 25,000)	The group containing AFB1 led to a relative abundance reduction of *Lactobacillus-aviarius*, *L.* *agilisand,* and *L.salivarius*; while an increase in *Staphylococcus-xylosus* and *Esherichia-coli-g-Escherichia-Shigella* compared with the control	AFB1 caused steatosis and inflammatory response in the liver, but no significant weight changes were observed. Also, AFB1 exposure decreased CAT, SOD, and GSH-Px activities, while MDA was significantly higher in contrast to control	Guo et al. 2023
**Mammals**							
32 C57BL/6 male mice six-weeks-old	3,830 µg/kg feed	2 weeks (Sub-chronic)	Colon	Biochemical analysis, histological analysis of tissues, and 16S rRNA sequencing of gut microbiota through V3 and V4 hypervariable regions	At phylum level, AFB1 increased Firmicutes and decreased Bacteroidetes. The relative abundance of *Clostridium* and *Lactobacillus* was enhanced in the AFB1 exposure, but *Akkermansia* was reduced compared to the control	AFB1 enhanced the serum levels of ALT, AST, ALP, and LPS regarding the control	Liu et al. 2022
10 male mice C57BL/6 J about 8 weeks old	6000 µg/kg feed	2 weeks (Sub-chronic)	Feces	Serum biochemistry, ELISA, gene expression analysis, western blotting, and gut microbiota analysis by 16 s rRNA gene amplicon sequencing through V4 hypervariable region	At the phylum level, the number of Bacteroidetes and Verrucomicrobia was reduced, but the number of Firmicutes was increased in the AFB1. The relative abundances of *Prevotella, Blautia, Akkermansia* and *Bacteroides* were decreased, but *Clostridium_XlVa, Bifidobacterium, Lactobacillus, Corynebacterium, Ruminococcus, Allobaculum* and *Coprococcus* abundance was increased in AFB1 exposure in contrast to control	Serum ALT, AST, ALP, and ROS levels were increased in AFB1-exposed mice. AFB1 also caused liver swelling and necrosis, severe diffuse bile duct hyperplasia, and inflammatory infiltration compared with the control	Liu et al. 2023
160 42-day-old weaned piglet	750 µg/kg feed	8 weeks (Sub-chronic)	Cecum	Histopathological and hematological analysis, gene expression level analysis, analysis of intestinal microbiota through 16 s rDNA sequencing through V3 and V4 hypervariable regions	At the phylum level, AFB1 increased Firmicutes and decreased Bacteroidetes. AFB1 led to an increase in the relative abundance of *Lactobacillus* and *Clostridium*, while a decrease in *Desulfovibrio, Alistipes* and *Lachnospiraceae* compared with the control	AFB1 resulted in an increase in liver/body weight and the enzymes ALT, ASP, and ALP. Also, AFB1 exposure decreased SOD and GSH-Px, but increased MDA, IL-6, and TNF-α in the serum in contrast to the control	Xu et al. 2023a, b
32 C57BL/6 J male mice 5-weeks-old	111 µg/kg feed	4 weeks (Sub-chronic)	Cecum	Histological and biochemical analysis and microbiota analysis were conducted through the bacterial 16S rRNA gene amplified by PCR	The AFB1 exposure reduced Bacteroidetes and *Cyanobacteria*, but increased Firmicutes and Proteobacteria. AFB1 led to an increase in the relative abundance of *Parabacteroides*, *Bacteroides*, *Escherichia-Shigella, Lactobacillus*, and a decrease of the relative abundance of *Prevotella* compared with control	AFB1 group showed significant induction in the weight of the liver and led to extensive inflammatory infiltrate and hepatocyte hypertrophy. Furthermore, AFB1 enhanced liver and serum LPS, NLRP3, caspase-1 and serum ALT, ASP, LDH, and MPO regarding the control	Ye et al. 2024
40 35-day-old rabbits	2,700 µg/kg feed	3 weeks (Sub-chronic)	Cecum	Serum biochemical analysis, histopathological assessment, antioxidant status of liver tissue and gut microbiota analysis through V3 and V4 hypervariable regions	At the phylum level, AFB1 increased Bacteroidetes and decreased Firmicutes in contrast to the control	Serum levels of ALT, AST, and ALP were significantly elevated in the AFB₁-treated group compared to the control group. Also, AFB1 caused liver damage regarding the control	Kong et al. 2025
32 male Sprague Dawley rats 7–8 weeks old	5–25 μg/kg feed	4 weeks (Sub-chronic)	Feces	Determination of liver weight and fecal bacterial profile	At the genus level, AFB1 exposure increased *Bifidobacterium*. In the case of *Escherichia*, low-AFB1 led to an increase, while high-AFB1 showed the opposite trend in comparison to the control. At the species level, *Lactobacillus spp.* was significantly lower following AFB1 exposure	Both low and high-dose AFB1 groups had significantly higher liver-to-body weight ratio than control group	Subramaniam et al., 2018
40 male Kunming mice 4 weeks old	6,333 µg/kg feed	24 h (Acute)	Feces	Serum biochemistry, serum antioxidant activities, and 16S rRNA sequencing through V3 and V4 hypervariable regions	Firmicutes, *Spirochaetes*, and Proteobacteria in the control exposure were lower than that in the AFB1 one, while the phylum Bacteroidetes and Actinobacteria in the control was higher than that in the AFB1. At the genus level, AFB1 increased *Lactobacillus*, *Bifidobacterium*, *Prevotella* and reduced *Blautia* comparing with the control	Compared with the control group, serum TG, TBIL, AST, ALT, and CRE in the AFB1 group all showed an increasing trend	Cao et al. 2021
12 sheep	25,000 µg/kg feed	24 h (Acute)	Feces	Serum biochemistry, liver histopathology, detection of apoptosis, liver function of tissue, liver antioxidant abilities, and Illumina sequencing through V3 and V4 hypervariable regions	Firmicutes*, Spirochaetes,* Verrucomicrobia, and Proteobacteria were more abundant in the AFB1 exposure, while Bacteroidetes were more abundant in the control one. AFB1 caused a relative abundance increase of *Clostridium,* while *Roseburia, Coprococcus, Prevotella* and *Butyrivibrio* decreased compared with the control	AFB1 exposure had significantly higher levels of IL-1β, IL-6, MDA, ALT, AST, and ALP expression than those of the control, but IL-10 and SOD expression were considerably lower in the AFB1. Also, abnormal hepatic lobule structure, disorganized hepatic cord arrangement, swollen and unevenly sized hepatocytes, severe infiltration of inflammatory cells, vacuolar degeneration, and necrosis were observed in AFB1 exposure in contrast to control	Sui et al. 2022
10 6-month-old healthy sheep	33,300 µg/kg feed	No available	Feces	Biochemistry analysis, 16S rRNA gene sequencing, and differential microbiota analysis through V3 and V4 hypervariable regions	At the phylum level, AFB1 led to a higher relative abundance of Bacteroidetes, Proteobacteria, Actinobacteria and Verrucomicrobia*,* but a decrease in Firmicutes. Also, compared with the control, the abundance of *Clostridium and Bifidobacterium* in the AFB1 exposure was increased, while *Prevotella* was reduced compared with the control	The activities of ALT and AST in the AFB1 exposure were elevated in comparison to the control	Lin et al. 2023
**Fish**							
20 Gibel carp	50–100 µg/kg feed	4 weeks (Sub-chronic)	Intestine	Liver antioxidant index, Illumina miSeq sequencing, and bioinformatics analysis through V3 and V4 hypervariable regions	At the phylum level, the AFB1 exposed groups showed a significantly higher abundance of Proteobacteria and Bacteroidetes and a significantly lower abundance of Firmicutes and Actinobacteria than the control. At the *genus* level, the AFB1 exposed groups showed a lower abundance of *Bacteroides* and *Clostridium* in comparison to the control	AFB1 exposed groups showed significantly higher ROS and MDA levels and significantly lower SOD levels in the liver than those in the control	Xue et al. 2023
480 juvenile turbot	100 µg/kg feed	8 weeks (Sub-chronic)	Intestine	Serum biochemical analysis, intestinal DNA extraction, and sequencing of intestinal microbiota by 16 s rRNA gene through V4 hypervariable region	AFB1 inclusion led to an increase in Firmicutes and a decrease in Proteobacteria and Bacteroidetes at the phylum level. The relative abundance of *Bifidobacterium, Stenotrophomonas, Paeniclostridium, Catonella, Agathobacter, Dorea, Faecalibaculum, Anaerostipes* of the AFB1 exposure were lower than in the control	AFB1 exposure decreased SOD and CAT in the liver, but increased MDA. On the other hand, AFB1 significantly upregulated the gene expression of p53, Bax, caspase3, caspase7, and caspase9 compared with control	Zhang et al. 2023
480 juvenile *Lateolabrax maculatus*	100–1,000 µg/kg feed	8 weeks (Sub-chronic)	Feces	Serum biochemical analysis and microbiota analysis were conducted through the bacterial 16S rRNA amplified by PCR	AFB1 increased the abundance of *Enterobacter,* but decreased the abundance of *Plesiomonas* regarding the control	AST, DAO, LPS, and DLA were linearly enhanced as dietary AFB1 increasing in comparison to the control	Peng et al. 2022

Antibiotic mixture *(ABX*). Aflatoxin B1 (*AFB1*). Alkaline phosphatase (*ALP*). Alanine transaminase (*ALT*). Arthrospira platensis (*AP*). Aspartate aminotransferase (*AST*). Basal diet pelleted at 75º (*C75*). Body weight (Body weight). Control diet at 75º with AFB1 degrader (C75 + DE). C75 supplemented with 0.5 g kg − 1 DE, pelleted at 75 °C (*C75 + DE*). Control diet at 80º with AFB1 degrader (*C80 + DE*). C80 supplemented with 0.5 g kg − 1 DE, pelleted at 80 °C (C80 + DE). Colony forming units (*CFU*). Compound mycotoxin detoxifier (*CMD*). Compound probiotic 1 (CP1). Compound probiotic 2 (*CP2*). Compound probiotic 3 (*CP3*). Creatinine (*CRE*). Diamine oxidase (*DAO*). Heat-resistant aflatoxin-degrading enzyme (*DE*). D-lactate (*DLA*). Deoxyribonucleic acid (*DNA*). Enzyme-linked immunosorbent assay (*ELISA*). Gamma-glutamyl transferase (*GGT*). Glycine-β-muricholic acid (*Gly-β-MCA*). Glutathione (GSH). Glutathione peroxidase (*GSH-Px*). Oxidized glutathione (*GSSG*). Humic acids (*HAs*). Hematoxylin–eosin (*HE*). Interleukin-1β (*IL-1β*). Interleukin 6 (*IL-6*). Interleukin 8 (*IL-8*). Interleukin 10 (*IL-10*). Lactate dehydrogenase (*LDH*). *Lactobacillus agilisand (L. Agilisand)*. *Lactobacillus salivarius (L.salivarius)*. Lipopolysaccharide (LPS). Mouse lysozyme (*LZM*). C75 containing 22.8% moldy corn, pelleted at 75° (*M75*). M75 supplemented with 0.5 g kg − 1 DE, pelleted at 75º (*M75 + DE*). C80 containing 22.8% moldy corn, pelleted at 80° (M80). M80 supplemented with 0.5 g kg − 1 DE, pelleted at 80º (*M80 + DE*). Malondialdehyde (*MDA*). Mycofix Plus (*MF*). Myeloperoxidase (*MPO*). Melatonin (*MT*). Magnotox-alphaA (*MTA*). Magnotox-alphaB (*MTB*). NLR family pyrin domain containing 3 (*NLRP3*). Tumor suppressor protein (p53). Polymerase chain reaction (PCR). Penthorum chinense Pursh Compound Flavonoids (PCPCF). Reactive oxygen species (*ROS*). Superoxide dismutase (*SOD*). Total bilirubin (*TBLI*). Total flavonoids of Rhizoma Drynaria (*TFRD*). Triglyceride (*TG*). Tumor necrosis factor-α (*TNF-α*). Mycotoxin-degrading enzymes (*YEMDE*).

#### OTA, gut microbiota and liver

##### Hepatic oxidative stress and inflammatory injury

OTA exposure caused pronounced hepatocellular damage, reflected by significant increases in AST, ALT, ALP, MDA, ROS, and circulating LPS, accompanied by marked reductions in SOD, GSH-Px, and tight junction protein expression across poultry, rodents, and rabbits (Huang et al. 2023; Lin et al. 2023; Liu et al. 2022, 2023; Peng et al. 2022, 2025; Qing et al. 2022; Xue et al. 2023; Zhang et al. 2023). These biochemical alterations indicate severe oxidative stress, compromised antioxidant defenses, and disruption of hepatocyte membrane integrity. Histological findings of hepatocyte necrosis, vacuolar degeneration, and inflammatory cell infiltration further confirm that OTA induces liver injury through combined oxidative and inflammatory pathways.

##### OTA-induced gut microbiota dysbiosis

Concomitant alterations in microbial composition were substantial. At the phylum level, OTA exposure was associated with increased Proteobacteria and Firmicutes, together with marked reductions in Bacteroidetes, Verrucomicrobia, and Actinobacteria. At the genus level, decreases in *Akkermansia* and *Prevotella* were consistently observed (Guo et al. 2023; Huang et al. 2023; Liu et al. 2022, 2023; Peng et al. 2022, 2025; Qing et al. 2022; Xue et al. 2023; Zhang et al. 2023). The depletion of these taxa is particularly relevant, as they play key roles in maintaining intestinal mucus integrity, producing SCFAs, and regulating epithelial tight junctions.

##### Intestinal barrier dysfunction and endotoxemia

At a mechanistic level, OTA-induced dysbiosis appears to compromise intestinal barrier function, as evidenced by decreased tight junction protein expression, thereby facilitating LPS translocation into the systemic circulation (Liu et al. 2024). Elevated LPS levels can activate hepatic inflammatory signaling pathways, promoting the release of pro-inflammatory mediators and enhancing ROS generation and lipid peroxidation, which directly contributed to hepatocyte damage (Shen et al. 2021; Wu et al. 2025). Simultaneously, the reduction in SCFA-producing bacteria likely diminishes anti-inflammatory and antioxidant signaling in hepatocytes, further weakening cellular defenses against oxidative stress. This combination of increased inflammatory stimuli and reduced protective metabolites establishes a self-perpetuating cycle of barrier dysfunction, endotoxemia, oxidative stress, and liver injury.

##### Alterations in bile acid metabolism and mucus layer integrity

Moreover, Firmicutes expansion may alter bile acid metabolism, potentially exacerbating hepatocellular stress and inflammatory responses, while the loss of Verrucomicrobia, particularly *Akkermansia*, may impair mucus layer renewal and further aggravate barrier permeability (Chiantera et al. 2023; Pellegrino et al., 2023; Rodrigues et al. 2022; Termite et al. 2025). These interconnected processes highlight that OTA-induced hepatotoxicity is not solely a result of direct toxic effects on liver cells but is strongly amplified by microbial-derived signals and impaired host–microbial interactions along the gut–liver axis.

##### Dose–response considerations

Moreover, no uniform dose–response relationship was evident across escalating OTA levels, likely reflecting the heterogeneity of animal species, experimental settings, and exposure durations among studies. Conversely, the hepatic findings were more consistent, with all doses triggering oxidative stress and promoting liver injury.

##### Modulation of the gut–liver axis as a therapeutic approach

Importantly, probiotic and prebiotic interventions were shown to partially reverse these alterations by restoring microbial diversity, increasing SCFA production, reinforcing barrier integrity, and reducing systemic endotoxin levels. These improvements were accompanied by normalization of liver enzyme profiles and attenuation of histopathological lesions (Zhou et al. 2024), underscoring the therapeutic potential of targeting microbial dysregulation to mitigate OTA-induced liver injury. All information is collected in Table 2, Table 2S, Figs. 4S, 5S and 6S.

**Table 2:** Overview of key characteristics of studies examining the effects of OTA on the interplay between microbiota and liver disease. The table compiles information on animal models, administered doses, exposure durations, experimental approaches, and main outcomes, highlighting the variability in study designs and the diverse microbiota and hepatic responses reported in the literature

Animal	Dose administration	Exposure time	Sample location	Experimental assay	Results		Reference
					**Microbiota**	**Liver**	
**Birds**							
180 one-day-old Jingfen No. 1 commercial chicks	101 μg/kg feed	6 weeks (Sub-chronic)	Cecum	Analysis of biochemical parameters in serum, kidney and liver, and 16S rRNA gene sequencing of the gut microbiota	At the phylum level, OTA caused a Bacteroidetes increase and a Firmicutes decrease. At a genus level, OTA decreased *Barnesiella* and increased *Ruminococcus* compared with control	As for liver damage parameters, OTA enhanced ALP and ASP, but reduced ALT. Also, OTA increased ROS, GSH, GSSG, IL-6, IL-8 and TNF-α significantly, but decreased IL-10 and SOD in comparison to control. On the other hand, GSH-Px values were similar in both AFB1 exposure and control	Qing et al. 2022
30 male one-day-old Peking ducklings	19,600 µg/kg feed	2 weeks (Sub-chronic)	Feces	Biochemical analysis and 16S rRNA amplicon sequencing through V3 and V4 hypervariable regions	OTA significantly altered the microbiota in the cecum and enhanced the relative abundance of Bacteroidetes and *Bacteroides* compared with the control	OTA exposure increased the activities of serum AST and ALT and the levels of liver LPS, IL-1β, and IL-6, but reduced IL-10 regarding the control	Wang et al. 2019b
60 one-day-old male Cherry Valley ducklings	19,600 µg/kg feed	3 weeks (Sub-chronic)	Feces	Biochemical analysis and metagenomics of cecum microbiota through V3 and V4 hypervariable regions	At the phylum level, OTA increased Bacteroidetes and decreased Firmicutes. At the genus level, OTA enhanced *Bacteroides* and the species *Turicibacter sanguinis* regarding the control	OTA increased ALT, AST, LPS, TLR4, MyD88, TNF-α, IL-6 and IL-1β levels compared with the control	Xia et al. 2021
720 mixed-sex 1-day-old White Pekin ducklings	2000 µg/kg feed	3 weeks (Sub-chronic)	Cecum	Serum lipid metabolism and liver antioxidant indices and analysis of the cecal microbiota community by 16S rRNA analysis through V3 and V4 hypervariable regions	At the phylum level, the OTA exposure reduced Firmicutes and Actinobacteria and enhanced Bacteroidetes. At the genus level, OTA decreased *Blautia*, *Butyricicoccus,* and *Butyricimonas* in comparison to the control	OTA increased AST, ALT, TG, HDL and LDL. Also, OTA had the lowest SOD, CAT, GSH-Px activities and the highest MDA level regarding the control	Zhai et al. 2020
160 one-day-old Cherry Valley ducklings	20,800 µg/kg feed	3 weeks (Sub-chronic)	Feces	Biochemical analysis, histopathology and gut microbiota analysis through V3 and V4 hypervariable regions (SILVA database)	At the phylum level, OTA increased Bacteroidetes and reduced Verrucomicrobia. At the genus level, OTA augmented *Bacteroides, Blautia* and *Alistipes*, but diminished *Streptococcus* and *Ruminococcus* in comparison to control	OTA exposure increased the liver LPS levels and the infiltration of inflammatory cells into the liver compared with the control	Zhai et al. 2025
**Mammals**							
40 male C57BL/6 J mice, 7-weeks-old	40,000 µg/kg feed	3 weeks (Sub-chronic)	Feces	Biochemical analysis and microbial community analysis through V3 and V4 hypervariable regions	At the genus level, OTA increased *Lactobacillus* and *Ileibacterium*, but decreased *Bifidobacterium, Bacillus,* *Desulfovibrio* and *Romboutsia* regarding the control	OTA enhanced ALT, ALP, LPS and MDA, but reduced GSH-Px and SOD in contrast to the control	Du et al. 2022
56 5-week-old healthy male C57BL/6 mice	1,330–6,670 µg/kg feed	4 weeks (Sub-chronic)	Colon	Hepatic biochemical parameters and 16S rRNA sequencing through V3 and V4 hypervariable regions	At the phylum level, OTA low-dose reduced Firmicutes, while increased Bacteroidetes, Proteobacteria, Actinobacteria and Verrucomicrobia. However, OTA high-dose increased Bacteroidetes and Actinobacteria, but decreased Proteobacteria. At the genus level, OTA decreased *Alloprevotella* and *Dubosiella*, but increased *Lactobacillus* compared with the control	Liver weight was increased by OTA-high dose, but not by low-dose. TC and TG levels were reduced by OTA-low dose, but increased by OTA-high dose in comparison to the control	Wang et al. 2024b
30 C57BL/6 male mice 5-weeks-old	1,100 µg/kg feed	3 weeks (Sub-chronic)	Colon	Biochemical and intestinal microbiota analysis through V3 and V4 hypervariable regions	At the phylum level, OTA significantly reduced the relative abundance of Bacteroidetes*,* Proteobacteria and Actinobacteria*,* while significantly increasing Firmicutes*.* At the genus level, OTA enhanced *Lactobacillus* and *Desulfovibrio*, but decreased *Bacteroides* and *Alloprevotella* regarding the control	In comparison with control, antioxidant activities of SOD, T-AOC and GSH-Px were lower, while IL-6, IL-1β, LPS, TNF-α, and MDA were greater in the liver OTA. Also, ALP, GGT, LDL, and TG were enhanced by OTA compared with the control	Zhang et al. 2021
120ICR mice 7-week-old	16,000 µg/kg feed	4 weeks (Sub-chronic)	Cecum	Liver function and gut microbiota analysis through V3 and V4 hypervariable regions	At the phylum level, OTA enhanced Bacteroidetes and Proteobacteria, but decreased Firmicutes and Verrucomicrobia. At the genus level, OTA decreased the relative abundance of *Lactobacillus* and *Akkermansia*, but increased *Helicobacter*, *Bacteroides*, *Parabacteroides* and *Mucispirillum* compared with the control	OTA decreased ALT, but enhanced AST liver activity. Also, TNF-α, IL-6 and IL-1β mRNA concentrations were significantly up-regulated in the liver of OTA-treated mice in comparison to the control	Yang et al. 2023a, b

Antibiotics (*ANTI*). Alkaline phosphatase (*ALP*). Alanine transaminase (*ALT*). Aspartate aminotransferase (*AST*). Body weight (Body weight). Catalase (*CAT*). Curcumin (*CUR*). Glutathione peroxidase (*GSH-Px*). Gamma-glutamyl transferase (*GGT*). Glutathione peroxidase (*GSH-Px*). High-density lipoprotein (*HDL*). High walnut green husk polysaccharides (*H-WGP*). Interleukin-1β (*IL-1β*). Interleukin 6 (*IL-6*). Interleukin 10 (*IL-10*). Low-density lipoprotein (*LDL*). Lipopolysaccharide *(LPS*). Low walnut green husk polysaccharides (*L-WGP*). Malondialdehyde (*MDA*). Melatonin (*MEL*). Myeloid differentiation primary response 88 (*MYD88*). Medium walnut green husk polysaccharides (*M-WGP*). Ochratoxin A (*OTA*). Ochratoxin-high (*OTA-H*). Ochratoxin-low (*OTA- L*). Total Antioxidant Capacity (*T-AOC*). Tibetan kéfir (*TK*). Superoxide dismutase (*SOD*). Triadimefon-high (*TDF-H.*) Triadimefon-low (*TDF-L*). Triglyceride (*TG*). Toll-like receptor 4 (*TLR4*). Tumor necrosis factor-α (*TNF-α*)

#### DON, gut microbiota and liver

## Hepatic oxidative stress and structural damage.

DON exposure induced pronounced hepatic oxidative injury, characterized by elevated ALT, AST, ALP, MDA, and ROS, together with reduced SOD, GSH-Px, and tight junction protein expression in poultry, rodents, and fish (Huang et al. 2023; Jia et al. 2024; Lin et al. 2023; Liu et al. 2022, 2023; Peng et al. 2022; Qing et al. 2022; Tao et al. 2023; Xu et al. 2023a, b; Ye et al. 2024). These biochemical changes reflect compromised hepatocyte membrane integrity, impaired antioxidant defenses, and enhanced lipid peroxidation. Histopathological observations of necrosis, vacuolar degeneration, and inflammatory cell infiltration further support the involvement of oxidative stress and inflammatory responses in DON-induced hepatotoxicity.

## DON-induced gut microbiota dysbiosis.

Concomitant alterations in microbial composition were dominated by an expansion of Proteobacteria and a reduction in Bacteroidetes, with variable shifts in Firmicutes, Actinobacteria, and Verrucomicrobia. At the genus level, increases in *Clostridium, Escherichia*, and *Lactobacillus* were accompanied by marked decreases in *Roseburia, Akkermansia,* and *Prevotella* (Cao et al. 2021; Chung et al., 2020; Guo et al. 2023; Huang et al. 2023; Liu et al. 2022, 2023; Peng et al. 2022, 2025; Qing et al. 2022; Tao et al. 2023; Xu et al. 2023a, b). The depletion of key SCFA–producing genera likely reduced anti-inflammatory signaling and weakened epithelial barrier integrity, thereby facilitating the translocation of microbial-derived endotoxins.

## Gut–liver axis–mediated mechanisms of injury.

At a mechanistic level, DON-induced dysbiosis appears to intensify LPS-driven hepatic inflammation, as increased endotoxin translocation can activate inflammatory signaling cascades in the liver, leading to enhanced ROS production and lipid peroxidation (Rizzatti et al. 2017; Vasques-Monteiro et al. 2021). Simultaneously, reduced SCFA availability may impair antioxidant defenses and mitochondrial function in hepatocytes, further exacerbating oxidative stress and promoting hepatocellular damage (Precup & Vodnar, 2019; Shen et al. 2021). Barrier dysfunction, reflected by decreased tight junction protein expression, likely reinforces this pathological loop by allowing continuous endotoxin influx, sustaining chronic inflammation and progressive liver injury (Yeoh et al., 2022).

## Dose–effect assessment

Regarding dose–response considerations, no uniform trend was observed across increasing DON doses, likely due to the heterogeneity in animal species, experimental designs, and exposure durations among the included studies. In contrast, hepatic outcomes showed greater consistency, as all tested doses were associated with oxidative stress and indicators of liver injury.

## Modulation of the gut–liver axis as a mitigation strategy.

Intervention studies support this integrative model, as supplementation with probiotics and dietary antioxidants restored microbial balance, enhanced SCFA production, reduced endotoxin burden, and attenuated oxidative stress, leading to improvements in liver enzyme profiles and histopathological features (Lin et al., 2020; Qiu et al., 2021; Yu et al. 2025). Together, these findings indicate that DON-induced hepatotoxicity arises not only from direct toxic effects on the liver but also from microbial dysregulation and barrier dysfunction, underscoring the central role of gut–liver axis interactions in mediating DON toxicity. All information is collected in Table 3, Table 3S, Figs. 7S, 8S and 9S.

**Table 3:** Overview of key characteristics of studies investigating the effects of DON on the interplay between microbiota and liver disease. The table summarizes animal models, administered doses, exposure durations, experimental approaches, and main findings, highlighting the variability in study designs and the observed effects on microbiota composition and liver outcomes

Animal	Dose administration	Exposure time	Sample location	Experimental assay	Results		Reference
					**Microbiota**	**Liver**	
**Birds**							
144 one-day-old Lingnan yellow-feathered male broilers	3 mg/kg feed	3 weeks (Sub-chronic)	Cecum	Oxidative status, gene expression and high-throughput sequencing of gut microbiota through V3 and V4 hypervariable regions	At the phylum level, DON exposure was enriched with Proteobacteria and depleted of Firmicutes*.* At the genus level, DON enhanced the relative abundance of *Escherichia/Shigella* regarding the control	DON increased ROS, O_2_^−^ MDA, CAT, and GSH-Px, but decreased T-AOC compared with the control	Wang et al. 2022b
Thirty-six male one-day-old Pudong Sanhuang broiler chickens	5–10 mg/kg feed	8 weeks (Sub-chronic)	Jejunum	Biochemical analysis and sequencing of 16 S rRNA of intestinal bacteria through V3 and V4 hypervariable regions	At the phylum level, DON increased the relative abundance of Firmicutes and Actinobacteria and decreased the relative abundance of Bacteroidetes and Proteobacteria*.* At the genus level, *Bifidobacterium, Escherichia-Shigella,* and *Romboutsia* relative abundance was increased, but that of *Bacteroides* was decreased after DON feeding compared with the control	H-DON increased the mean levels of ALT, TBIL, and UA, but decreased the mean levels of ALB and TG in comparison to the control	Jia et al. 2023
**Mammals**							
Forty 8-week-old male C57BL/6 J mice	179 µg/kg feed	30 days (Sub-chronic)	Feces	Biochemical parameters, histopathological analysis and 16S rRNA gene sequencing	At the phylum level, DON enhanced Proteobacteria and Verrucomicrobia, but decreased Firmicutes, Actinobacteria and Bacteroidetes. At the genus level, *Parabacteroides*, *Enterobacter* and *Akkermansia* displayed the most increase in abundance after DON administration while *Lactobacillus, Odoribacter*, *Prevotella* and *Lachnospiracea* showed the greatest decline in contrast to the control	Low-grade lymphocyte infiltration around hepatic centrilobular veins emerged in DON exposure. Also, ALT and AST levels were increased by DON compared with the control	Peng et al. 2019
144 three-week-old Balb/C mice	33 mg/kg feed	2 weeks (Sub-chronic)	Colon	Biochemical analysis, histopathological examination and microbiota analysis through V3 and V4 hypervariable regions	At the phylum level, DON reduced the abundance of Bacteroidetes and Actinobacteria and increased that of Firmicutes*.* At the genus level, DON enhanced *Lactobacillus* and *Turicibacter,* but reduced *Ruminococcus* in contrast to the control	DON increased serum AST and ALT levels in a significant manner. In addition, DON induced inflammatory infiltration and hyaline degeneration compared with the control	Wang et al. 2020a, b
20 female, 21-day-old piglets	3.5 mg/kg feed	3 weeks (Sub-chronic)	Cecum	Serum biochemical parameters, oxidative stress and cecal microbiota population analysis	At the phylum level, Firmicutes was decreased by DON. At the genus level, DON enhanced *Escherichia*, but decreased *Lactobacillus* in comparison to the control	DON augmented ALP and AST levels, but diminished ALT. Also, DON exposure significantly increased the concentrations of MDA in the serum and H_2_O_2_ level in the liver; while there was a significant decrease in levels of GSH-Px in the liver regarding the control	Jia et al. 2021
Thirty-two crossbred piglets, 28–29 weeks old, mixed sex	2524 μg/kg feed	2 weeks (Sub-chronic)	Feces	Histological, biochemical and metagenomic analysis	At the phylum level, DON decreased Proteobacteria and Firmicutes, but enhanced *Ascomycota*. At the species level, *S. cerevisiae* and *F. prausnitzii* were reduced by DON, but *Aureobasidium namibiae, L. mucosae* and *L. amylorovus* were increased by DON compared with the control	DON reduced ALP, ALT and AST serum levels, but increased ALB, CREA1 and urea in comparison to control. Also, DON caused moderate to severe vacuolation of hepatocytes and megalocytosis, apoptosis and focal necrosis regarding the control	Novak et al. 2021
160 42-day-old weaned piglets	1040 µg/kg feed	4 weeks (Sub-chronic)	Feces	Biochemical analysis and 16S rRNA gene sequencing analysis	At the phylum level, DON increased Bacteroidetes*,* Actinobacteria and Proteobacteria and decreased Firmicutes*.* At the genus level, the relative abundances of *Blautia* and *Megasphaera* were significantly increased, whereas *Subdoligranulum, Clostridium*, *Ruminococcus* and *Lactobacillus* were decreased regarding the control	LDH content, ALT and AST activity in DON-challenged exposure was significantly increased, compared with the control	Xu et al. 2023b
Twenty-eight 28-d-old barrows	4 mg/kg feed	4 weeks (Sub-chronic)	Feces	Biochemical parameters, histopathological analysis and 16S rRNA gene sequencing	At the phylum level, Bacteroidetes and Actinobacteria were decreased by DON exposure, while Firmicutes was enhanced. At the genus level, DON reduced *Lactobacillus*, *Blautia* and *Enterorhabdus* compared with the control	DON led to higher serum concentrations of ALP, ALT and AST. Also, Ingestion of DON alone caused focal necrosis, hepatocyte cytoplasmic vacuolization and degeneration, as well as hepatic cord disorders regarding the control	Wang et al. 2020b
Twelve conventional Shanghai White pigs	10 mg/kg feed	8 weeks (Sub-chronic)	Jejunum	Biochemical analysis and sequencing of 16 S rRNA of jejunum intestinal bacteria through V3 and V4 hypervariable regions	At the phylum level, DON increased the relative abundance of Firmicutes and decreased the relative abundance of Bacteroidetes*.* At the genus level, DON significantly decreased the relative abundance of *Streptococcus* and *Prevotella* in contrast to the control	H-DON increased the mean levels of AST, TBIL, and CREA1, but decreased the mean levels of ALP and UA in comparison to the control	Jia et al. 2023

Albumin (ALB). Alkaline phosphatase (ALP). Alanine transaminase (ALT). Aspartate aminotransferase (AST). Beauvericin (BEA). Catalase (CAT). Creatinine 1 (CREA1). Deoxynivalenol (DON). DON with catalase (DONC). Enniatin B1 (ENNB). Enniatin B1 (ENNB1). Faecalibacterium prausnitzii (F. prausnitzii). Glycyrrhizic acid combined with compound probiotics (GAP). Glutathione peroxidase (GSH-Px). High deoxynivalenol (H-DON). Hydrogen peroxide (H_2_O_2_). Heme oxygenase-1 (HO-1). Lactobacillus (L). Low deoxynivalenol (L-DON). Malondialdehyde (MDA). Superoxide ion (O_2_^–^). Reactive oxygen species (ROS). Sodium butyrate (SB). Total bilirubin (TBLI). Total Antioxidant Capacity (T-AOC). Triglyceride (TG). Uric acid (UA). Bacillus licheniformis strain (YB9)

### ZEN, fumonisins (FBs), ENNB + ENNB1 + BEA and T-2, gut microbiota and liver

Zearalenone (ZEN), fumonisins (FBs), T-2 toxin, and enniatins (ENNB, ENNB1, and beauvericin [BEA]) induce significant hepatotoxicity across multiple animal species, with mounting evidence indicating that their adverse hepatic effects are strongly modulated by gut microbiota dysregulation and impaired gut–liver axis signaling. Similar to AFB1, OTA, and DON, these mycotoxins exert combined direct hepatic toxicity and indirect effects mediated by microbial-derived inflammatory and metabolic signals (Bertero et al., 2020; Huang et al. 2023; Lin et al. 2023; Liu et al. 2022, 2023; Peng et al. 2022, 2025; Xue et al. 2023; Zhang et al. 2023).

## Hepatic oxidative stress and inflammatory injury.

Exposure to ZEN, FBs, T-2 toxin, and ENNs consistently resulted in significant elevations of serum ALT, AST, and ALP, indicative of hepatocellular damage. These changes were accompanied by increased malondialdehyde (MDA), reactive oxygen species (ROS), and circulating lipopolysaccharide (LPS) levels, together with reductions in antioxidant enzymes such as superoxide dismutase (SOD) and glutathione peroxidase (GSH-Px). Concurrent decreases in tight junction protein expression further suggest compromised epithelial and hepatocyte membrane integrity. Histopathological findings—including hepatocyte necrosis, vacuolar degeneration, fatty infiltration, and inflammatory cell infiltration—confirm that oxidative stress and inflammation are central drivers of liver injury induced by these mycotoxins (Nagl et al. 2021).

## Gut microbiota alterations induced by ZEN, FBs, T-2, and ENNs.

In parallel with hepatic injury, exposure to these mycotoxins produced gut microbial alterations that largely mirrored those reported for AFB1, OTA, and DON. At the phylum level, a consistent expansion of Proteobacteria and Actinobacteria was observed, alongside reductions in Bacteroidetes and Verrucomicrobia. At the genus level, a marked depletion of *Akkermansia*, *Prevotella*, and *Roseburia* was repeatedly reported (Guo et al. 2023; Huang et al. 2023; Liu et al. 2022, 2023; Peng et al. 2022, 2025; Xue et al. 2023). The loss of these taxa is particularly consequential given their central roles in short-chain fatty acid (SCFA) production, mucus layer maintenance, and regulation of epithelial tight junctions.

## Gut–liver axis–mediated mechanisms of hepatotoxicity.

The depletion of SCFA-producing and barrier-supportive bacteria likely weakens intestinal barrier integrity, facilitating translocation of microbial-derived LPS into the circulation, as reflected by elevated systemic LPS levels in exposed animals. Increased LPS activates hepatic inflammatory signaling pathways, thereby amplifying ROS generation, lipid peroxidation, and inflammatory cell recruitment. These processes synergistically contribute to the observed elevations in liver enzymes and histopathological damage. Simultaneously, reduced SCFA availability may impair anti-inflammatory signaling and antioxidant capacity in hepatocytes, further exacerbating oxidative stress and limiting hepatic regenerative responses. Collectively, these findings indicate that microbial dysbiosis is not merely a secondary consequence of toxicity but an active contributor to the progression of liver injury through reinforcement of inflammatory and oxidative pathways along the gut–liver axis.

## Dose-related effects

In terms of dose–response, no consistent escalation in effects with higher doses was identified, likely due to study heterogeneity. Nonetheless, all doses consistently induced gut microbiota changes, oxidative stress and liver injury.

## Microbiota-targeted interventions and mitigation strategies.

Intervention studies provide compelling evidence for the therapeutic potential of targeting the gut–liver axis to mitigate hepatotoxicity induced by ZEN, FBs, T-2 toxin, and ENNs. Postbiotic supplementation, including SCFAs and yeast cell wall extracts (YCWE), partially reversed toxin-induced alterations by improving intestinal barrier integrity, reducing oxidative stress, and attenuating liver injury (Saleemi et al. 2023; Yu et al. 2025; Choi et al. 2025). SCFAs—particularly butyrate—have been shown to limit endotoxin translocation and suppress hepatic inflammation, while YCWE reduces mycotoxin bioavailability through toxin binding (Albillos et al. 2020; Kim et al. 2019; Kim & Yang 2024; Rose et al. 2018).

YCWE components, including β-glucans and mannan-oligosaccharides, bind Fusarium-derived mycotoxins via hydrogen bonding and van der Waals interactions, forming complexes that are excreted in feces and thereby preventing systemic absorption (Yu et al. 2025). By reducing luminal toxin exposure, YCWE indirectly supports intestinal barrier integrity, as evidenced by increased expression of tight junction proteins (e.g., occludin, claudin-4, and ZO-1) and improvements in gut morphology and inflammatory markers.

Similarly, probiotic and prebiotic interventions enhanced antioxidant defenses and SCFA availability, leading to improved liver enzyme profiles and reduced histopathological damage (Stojanov et al., 2019). Together, these findings underscore the central role of gut–liver axis interactions in ZEN-, fumonisin-, T-2 toxin-, and enniatin-induced hepatotoxicity and support microbiota-targeted strategies as promising approaches to mitigate mycotoxin-induced liver damage (Ben Salah-Abbès et al. 2020; Guo et al., 2020; Mandal et al. 2024; Senchukova 2023; Shen et al. 2021; Wu et al. 2025). A detailed synthesis of these data is provided in Table 4, while Table 5 summarizes the effects of mycotoxins on bacterial genera.

**Table 4:** Overview of key characteristics of studies examining the effects of ZEN, FUM, BEA, ENNs, and T-2 on the interplay between microbiota and liver disease. The table summarizes animal models, administered doses, exposure durations, experimental approaches, and main findings, highlighting the heterogeneity in study designs and the reported impacts on microbiota and liver function

Animals	Dose administration	Exposure time	Sample location	Experimental assay	Results		Reference
					Microbiota	Liver	
Birds							
Six 42-day-old broilers	2,5 mg/kg feed	3 weeks (Sub-chronic)	Duodenum, ileum and cecum	Haematological and histopathological examination and cecum intestinal flora analysis through V3 and V4 hypervariable regions	At the phylum level, ZEN increased Firmicutes and decreased Bacteroidetes*.* At the genus level, ZEN enhanced *Romboutsia* and *Enterococcus,* but decreased the relative abundance of *Streptococcus* compared with the control	ZEN increased MDA and T-SOD and decreased GSH, SOD, T-AOC. ZEN led to obvious infiltration of inflammatory cells in liver tissues, peripheral oedema of blood vessels, vascular congestion in blood vessels, and slight circular vacuolation of cells in comparison to the control	Jia et al. 2022
30 male Pekin ducks, 1 day of age	2 mg/kg feed	2 weeks (Sub-chronic)	Ileum	Biochemical parameters and 16S rRNA gene sequencing through V3 and V4 hypervariable regions	At the phylum level, T-2 exposure augmented Firmicutes, but reduced Bacteroidetes and Proteobacteria. At the genus level, T-2 enhanced *Enterococcus, Rothia* and *Lactococcus*, but diminished *Corynebacterium, Gallicola, Leucobacter, Aerosphaera, Atopostipes, Jeotgalibaca, Actinomyces,* and *Turicibacter* regarding the control	T-2 exposure exhibited hepatic steatosis and inflammatory cell infiltration, elevated serum ALT and AST activities, increased hepatic inflammatory cytokines (TNF-α, IL-1β, IL-6 and IL-10), and upregulated TLR4 signaling and lipid related genes compared with the control	An et al. 2025
Mammals							
Six dry Holstein cows 10 years old	1000 mg/kg feed	2 days (Sub-chronic)	Stomach	Biochemical parameters and 16S rRNA gene sequencing through V3 and V4 hypervariable regions	At the phylum level, FBs reduced Bacteroidetes and increased Firmicutes*.* At the genus level, FBs increased *Coprococcus* and *Ruminiclostridium*, but decreased *Prevotella* and *Butyrivibrio* regarding the control	FBs increased ALP, AST, GLDH and GGT serum levels compared with the control	Hartinger et al. 2022
30 Six-week-old female Balb/c mice	30 mg/kg feed	6 weeks (Sub-chronic)	Feces	Serum biochemical analysis and 16S rRNA gene sequencing	At the phylum level, FB1 augmented Bacteroidetes and Actinobacteria, but reduced Firmicutes. At the genus level, FB1 increased *Lactobacillus, Dehalobacterium, Butyricimonas, Mucispirillum, Bacteroides, Ruminococcus, Prevotella, Coprococcus, Anaeroplasma, Adlercreutzia,* and *Alistipes*, while diminished *Oscillospira, Anaerostipes, Streptococcus, Roseburia,, Bilophila,* and *Turicibacter* compared with the control	FB1 exposure increased the levels of AST, ALP and ALT. Also, small round vacuoles were evident (black arrows) and focal inflammatory cell infiltration was observed regarding the control	Ye et al. 2025
40 BALB/c female mice, 3 weeks old	60 mg/kg feed	2 weeks (Sub-chronic)	Colon	Haematological and histopathological examination and intestinal flora analysis	At the phylum level, ZEN decreased Firmicutes and Actinobacteria, but augmented Bacteroidetes and Proteobacteria*.* At the genus level, ZEN reduced *Ruminococcus, Akkermansia* and *Roseburia,* but enhanced *Turicibacter* and *Bacteroides* compared with the control	ZEN increased the weight of the liver and the serum levels of AST and ALT in comparison to the control	Wang et al. 2018
24 4-week-old SPF-grade C57BL/6 J male mice	200–2,000 µg/kg feed	12 weeks (Sub-chronic)	Feces	Blood biochemistry tests, determination of liver tissue and gut microbiota analysis	At the phylum level, ZEN augmented Firmicutes, but decreased Bacteroidetes and Actinobacteria regarding the control	ZEN enhanced hepatocyte swelling, cytoplasmic vacuolisation, contraction of the nucleoli, loss of normal morphology of liver lobules, and a significant increase in the number and area of fat vacuoles and a large amount of lipid deposition compared with the control	Han et al. 2025
Thirty-two 5-week-old C57BL/6 J male mice	1.4 mg/kg feed	9 weeks (Sub-chronic)	Colon	Serum biochemical indicators and 16 s rRNA gene sequencing	At the phylum level, ZEN augmented Proteobacteria, but diminished Firmicutes and Verrucomicrobia. At the genus level, the abundance of *Enterobacter, Escherichia_Shigella, Bacteroides,* and *Salmonella* was increased, while that of *Akkermansia* was decreased in contrast to the control	ZEN exposure caused lipid metabolism disorders by increasing the LDL levels. Also, ZEN augmented serum AST levels compared with the control	Wang et al., 2022c
32female rats	92.3 mg/kg feed	4 weeks (Sub-chronic)	Cecum	Serum biochemical indicators and 16 s rRNA gene sequencing through V1-V9 hypervariable regions	At the phylum level, ZEN exposure reduced Proteobacteria and Verrucomicrobia. At the genus level, ZEN augmented *Clostridium*, but diminished *Lactobacillus* regarding the control	ZEN disrupted enterohepatic circulation of bile acids—increase in total bile acids, a rise in a specific bile acid (glycochenodeoxycholic acid), and a shift in the ratio of conjugated to unconjugated bile acids	Yuan et al. 2024
32 crossbred piglets, 28–29 weeks old, mixed sex	2524 μg/kg feed	2 weeks (Sub-chronic)	Feces	Histological, biochemical and metagenomic analysis	At the phylum level, ENNB + ENNB1 + BEA led to a relative abundance increase in Firmicutes and Actinobacteria, while a reduction in *Ascomycota* and Proteobacteria*.* At the species level, *L. reuteri*, *L. mucosae* and *Mycobacterium branderi* were enhanced by ENNB + ENNB1 + BEA, but *L. amylorovus* and *E. coli* were increased by ENNB + ENNB1 + BEA in contrast to the control	ENNB + ENNB1 + BEA caused an increase in ALP, but a decrease in ALT and AST. Also, ENNB + ENNB1 + BEA exerted moderate to severe vacuolation of hepatocytes and megalocytosis compared with the control	Novak et al. 2021

Alkaline phosphatase (*ALP*). Alanine aminotransferase (*ALT*). Aspartate aminotransferase (*AST*). *Bacillus cereus strain* (*BC7*). Beauvericin (*BEA*). Deoxynivalenol (*DON*). Enniatin B (Enniatin B). Enniatin B1 (ENNB1). Fumonisin (*FBs*). γ-glutamyl-transferase (*GGT*). Glutamate dehydrogenase (*GLDH*). Glutathione (*GSH*). High fat diet (*HFD*). *Lactobacillus amylorovus (L. amylorovus). Lactobacillus mucosae (L. mucosae). Lactobacillus reuteri (L. reuteri).* Malondialdehyde (*MDA*). Superoxide dismutase (SOD). Zearalenone (*ZEN*).

**Table 5:** Effects of different mycotoxins on animal microbiota at the bacterial genus level. The table highlights which genera were most frequently affected, indicating patterns of microbial alterations associated with mycotoxin exposure

Mycotoxin	Effect on bacterial genus		
	**Increase**	**Decrease**	**No effect**
AFB1	*Bifidobacterium, Lactobacillus, Clostridium, Ruminococcus, Escherichia, Bacteroides, Allobaculum, Staphylococcus,* and *Corynebacterium*	*Prevotella, Akkermansia* and *Roseburia*	*Blautia,* and *Coprococcus*
OTA	*Lactobacillus, Bacteroides, Blautia,* and *Turicibacter*	*Bifidobacterium, Akkermansia,* and *Alloprevotella*	*Ruminococcus*
DON	*Bifidobacterium, Akkermansia, Escherichia*, and *Turicibacter*	*Lactobacillus, Clostridium, Ruminococcus, Bacteroides,* and *Prevotella*	*Blautia*
ZEN	*Turicibacter, Bacteroides, Romboutsia,* and* Enterococcus*	*Akkermansia, Roseburia, Streptococcus, Butyrivibrio,* and *Pseudobutyrivibrio*	*Ruminococcus*
FBs	*Coprococcus* and* Ruminiclostridium,*	*Prevotella* and* Butyrivibrio*	
ENNB + ENNB1 + BEA	*L. reuteri, L. mucosae,* and* Mycobacterium branderi*	*L. amylorovus* and* E. coli*	
T-2	*Enterococcus, Rothia* and *Lactococcus*	*Corynebacterium, Gallicola, Leucobacter, Aerosphaera, Atopostipes, Jeotgalibaca, Actinomyces,* and* Turicibacter*	

AFB1 generally increases *Bifidobacterium*, *Lactobacillus*, *Clostridium*, *Ruminococcus*, and *Escherichia*, while reducing *Prevotella*, *Akkermansia*, and *Roseburia*. OTA exposure is associated with increased *Lactobacillus*, *Bacteroides*, and *Blautia*, but decreased *Bifidobacterium* and *Akkermansia*. DON displays a distinct profile, increasing *Bifidobacterium*, *Akkermansia*, and *Escherichia*, while reducing *Lactobacillus*, *Clostridium*, *Ruminococcus*, *Bacteroides*, and *Prevotella*. ZEN, fumonisins, enniatins, BEA, and T-2 toxin similarly disrupt SCFA-producing and barrier-supportive genera—particularly *Akkermansia*, *Roseburia*, *Butyrivibrio*, and *Prevotella*—highlighting a convergent pattern of dysbiosis linked to impaired intestinal integrity and inflammation.

Beyond compositional shifts, the gut microbiota contributes directly to mycotoxin detoxification through enzymatic biotransformation and adsorption. Several bacterial enzymes, including laccases, esterases, dehydrogenases, and aldo–keto reductases, metabolize mycotoxins or their conjugates (Abraham et al. 2022). For example, *Butyrivibrio fibrisolvens*, *Roseburia intestinalis*, and *Eubacterium rectale* hydrolyze glucosylated trichothecenes, whereas *Bifidobacterium adolescentis* and *Lactiplantibacillus plantarum* selectively metabolize DON-Glc (Daud et al. 2020). In parallel, bacterial components such as β-D-glucans effectively adsorb non-steroidal mycotoxins (e.g., ZEN), reducing intestinal bioavailability and promoting fecal excretion (Mirseyed et al. 2024).

Despite growing evidence linking mycotoxin-induced dysbiosis to liver injury, only a limited number of compounds (AFB1, OTA, DON, ZEN, FBs, enniatins, BEA, and T-2) have been evaluated within a gut–liver axis framework (Table 4S**;** Fig. 10S). Substantial heterogeneity in animal models, exposure conditions, and microbiota assessment methods further limits cross-study comparability (Table 5S**; **Fig. 11S). Moreover, most studies focus on taxonomic changes, while functional microbial outputs, including protective metabolites such as short-chain fatty acids and potentially harmful metabolites like trimethylamine, remain underexplored (Janeiro et al., 2018; Tan et al., 2019). Addressing these gaps through integrated compositional and functional analyses will be essential for elucidating microbiota-mediated mechanisms of mycotoxin toxicity and for developing targeted preventive strategies. A schematic overview is provided in Fig. 12S.

## Conclusions

Mycotoxins simultaneously disrupt gut microbiota composition and liver function, with microbial shifts—particularly depletion of SCFA-producing and barrier-supportive taxa (*Akkermansia, Roseburia, Prevotella*) and expansion of pro-inflammatory genera (Proteobacteria, *Escherichia*)—compromising intestinal integrity and facilitating endotoxin-driven hepatic oxidative stress and inflammation. These changes are reflected in elevated liver enzymes (ALT, AST, ALP), lipid peroxidation markers (MDA, ROS), inflammatory mediators (TNF-α, LPS), and reduced antioxidant defenses (SOD, GSH-Px). Microbiota-derived metabolites, such as protective SCFAs and potentially harmful TMA, further modulate liver injury. Despite over 400 identified mycotoxins, research has focused on a limited subset (AFB1, OTA, DON, ZEN, ENNs, BEA, FBs, T-2), and findings are often inconsistent due to variations in species, dose, exposure duration, and experimental design. These insights underscore the pivotal role of the gut–liver axis in mycotoxin toxicity and highlight the urgent need for integrated mechanistic studies to inform targeted preventive, therapeutic, and regulatory strategies.

## Supplementary Information

Below is the link to the electronic supplementary material.

ESM 1 (DOCX.0.97 MB)

## Data Availability

All data included in this scoping review are obtained from previously published sources, which are fully cited in the manuscript. No new primary data were generated. Extracted information, including study characteristics, key findings, and categorization details, is available from the corresponding author upon reasonable request.
