# Supplementary material for: Mycotoxins, gut microbiota alterations and liver disease in animals: A scoping review
Source: Cell Biol Toxicol. 2026 Feb 9;42(1):39. doi: 10.1007/s10565-026-10156-5 (PMC12945928; doi:10.1007/s10565-026-10156-5)
Supplement: Supplementary file 1 — (DOCX.0.97 MB) [file 10565_2026_10156_MOESM1_ESM.docx]

**Figure 1S.** Bar graph summarizing studies that report changes in gut microbiota at the phylum level following AFB1 (aflatoxin B1) exposure. The direction of change—either an increase or decrease in abundance—is based on the findings reported by the authors of each study. Data were collected from 20 publications (n=20), highlighting which microbial phyla are most commonly affected by AFB1.

**Figure 2S.** Bar graph illustrating the effects of ochratoxin A (OTA) on gut microbiota at the genus level. The graph shows the number of studies reporting **increases** or **decreases** for each genus, with the analyzed genera divided into two groups: **Group a:** *Blautia, Allobaculum, Escherichia, Ruminococcus, Clostridium, Lactobacillus,* and *Bifidobacterium*; **Group b:** *Coprococcus, Roseburia, Corynebacterium, Turicibacter, Bacteroides, Alloprevotella,* and *Staphylococcus*. The direction of change (increase or decrease) is based on the original authors’ reports, and a total of 20 publications (n = 20) were included in this analysis. This figure provides an overview of the variability and trends in microbiota alterations induced by OTA exposure at the genus level.

**Figure 3S.** Bar graph showing the liver parameters most frequently reported to be altered in animals exposed to aflatoxin B1 (AFB1). The graph displays the number of publications reporting changes in each parameter, with a total of 20 studies included (n = 20). Abbreviations: ALP, alkaline phosphatase; ALT, alanine transaminase; AST, aspartate aminotransferase; GSH-Px, glutathione peroxidase; Inf. infiltration, inflammatory infiltration; LPS, lipopolysaccharide; MDA, malondialdehyde; ROS, reactive oxygen species; SOD, superoxide dismutase. This figure highlights the variability and frequency of liver biomarker alterations associated with AFB1 exposure.

**Table 1S.** Summary of the frequency of studies according to the liver parameters evaluated in relation to the gut microbiota–liver axis. The parameters include ALP, ALT and AST as markers of liver function; MDA and ROS as indicators of oxidative stress; SOD and GSH-Px as key antioxidant enzymes; inflammatory infiltration as a histological marker of liver inflammation; and LPS as an inflammatory stimulus. This table highlights which liver-related biomarkers are most frequently assessed across the studies analyzed.

| **Liver parameter** | **Number of studies** |
| --- | --- |
| ALP/ALT/ASP | 14 |
| MDA/ROS | 10 |
| SOD/GSH-Px | 9 |
| Inflammatory infiltration | 5 |
| LPS | 3 |
| IL-1β/IL-6/IL-8/IL-10 | 3 |

ALP: Alkaline Phosphatase; ALT: Alanine Aminotransferase; AST: Aspartate Aminotransferase; GSH-Px: Glutathione Peroxidase; LPS: Lipopolysaccharide; MDA: Malondialdehyde; ROS: Reactive Oxygen Species; SOD: Superoxide Dismutase.

**Figure 4S.** Bar graph illustrating the effects of ochratoxin A (OTA) on gut microbiota at the phylum level. The graph shows the number of studies reporting **increases** or **decreases** for each phylum, with the direction of change based on the original authors’ reports. A total of 9 publications (n = 9) were included in this analysis. This figure provides an overview of the variability and trends in microbiota alterations at the phylum level induced by OTA exposure.

B)

**Figure 5S.** Bar graph illustrating the effects of ochratoxin A (OTA) on gut microbiota at the genus level. The graph shows the number of studies reporting **increases** or **decreases** for each genus, with the direction of change based on the original authors’ reports. The analyzed genera are divided into two groups: **Group a:** *Blautia, Allobaculum, Escherichia, Ruminococcus, Clostridium, Lactobacillus,* and *Bifidobacterium*; **Group b:** *Coprococcus, Roseburia, Corynebacterium, Turicibacter, Bacteroides, Alloprevotella*, and *Staphylococcus*. A total of 9 publications (n = 9) were included in this analysis. This figure provides an overview of the variability and trends in genus-level microbiota alterations induced by OTA exposure.

**Figure 6S.** Bar graph showing the liver parameters most frequently reported to be altered in animals exposed to ochratoxin A (OTA). The graph displays the number of publications reporting changes in each parameter, with a total of 9 studies included (n = 9). Abbreviations: ALP, alkaline phosphatase; ALT, alanine transaminase; AST, aspartate aminotransferase; GSH-Px, glutathione peroxidase; LPS, lipopolysaccharide; MDA, malondialdehyde; ROS, reactive oxygen species; SOD, superoxide dismutase; TNF-α, tumor necrosis factor-α. This figure highlights the variability and frequency of liver biomarker alterations associated with OTA exposure.

**Table 2S.** Summary of the frequency of studies according to the liver parameters evaluated in relation to the gut microbiota–liver axis. The parameters include ALP, ALT, AST as indicators of liver function; LPS as an inflammatory marker; SOD and GSH-Px as antioxidant enzymes; TNF-α as a pro-inflammatory cytokine; and MDA and ROS as markers of oxidative stress. This table highlights which liver-related biomarkers are most frequently assessed across the studies analyzed.

| **Liver parameter** | **Number of articles** |
| --- | --- |
| ALP/ALT/ASP | 7 |
| LPS | 5 |
| SOD/GSH-Px | 4 |
| TNF-α | 3 |
| MDA/ROS | 3 |

ALP: Alkaline Phosphatase; ALT: Alanine Aminotransferase; AST: Aspartate Aminotransferase; GSH-Px: Glutathione Peroxidase; LPS: Lipopolysaccharide; MDA: Malondialdehyde; ROS: Reactive Oxygen Species; SOD: Superoxide Dismutase; TNF-α: Tumor Necrosis Factor Alpha.

**Figure 7S.** Bar graph illustrating the effects of deoxynivalenol (DON) on gut microbiota at the phylum level. The graph shows the number of studies reporting increases or decreases for each phylum, with the direction of change based on the original authors’ reports. A total of 9 publications (n = 9) were included in this analysis. This figure provides an overview of the variability and trends in phylum-level microbiota alterations induced by DON exposure.

A)

**Figure 8S.** Bar graph illustrating the effects of deoxynivalenol (DON) on gut microbiota at the genus level. The graph shows the number of studies reporting increases or decreases for each genus, with the direction of change based on the original authors’ reports. The analyzed genera are divided into two groups: Group a: *Blautia, Allobaculum, Escherichia, Ruminococcus, Clostridium, Lactobacillus,* and *Bifidobacterium*; Group b: *Coprococcus, Roseburia, Corynebacterium, Turicibacter, Bacteroides, Alloprevotella,* and *Staphylococcus*. A total of 9 publications (n = 9) were included in this analysis. This figure provides an overview of the variability and trends in genus-level microbiota alterations induced by DON exposure.

**Figure 9S.** Bar graph representing the liver parameters most affected in animals exposed to DON, based on data from nine publications (n=9). The parameters measured include markers of liver function and oxidative stress: ALP, ALT and AST as indicators of liver injury; GSH-Px and SOD as key antioxidant enzymes; MDA and ROS as markers of oxidative damage; and LPS as an inflammatory stimulus. This figure highlights which liver-related biomarkers are most commonly altered following DON exposure in animal studies.

**Table 3S.** Summary of the frequency of studies according to the liver parameters assessed in relation to the gut microbiota–liver axis. The parameters include ALP, ALT, and AST as markers of liver function; SOD and GSH-Px as antioxidant enzymes; CREA1 as a metabolic indicator; hepatocyte vacuolization as a histological marker of liver injury; and MDA and ROS as indicators of oxidative stress. This table highlights which liver-related biomarkers are most frequently investigated across the studies analyzed.

| **Liver parameter** | **Number of studies** |
| --- | --- |
| ALP/ALT/ASP | 8 |
| SOD/GSH-Px | 2 |
| CREA1 | 2 |
| Hepatocyte vacuolization | 3 |
| MDA/ROS | 2 |

ALP: Alkaline Phosphatase; ALT: Alanine Aminotransferase; AST: Aspartate Aminotransferase; CREA: Creatinine; GSH-Px: Glutathione Peroxidase; MDA: Malondialdehyde; ROS: Reactive Oxygen Species; SOD: Superoxide Dismutase.

**Figure 10S.** Illustration of the distribution of various mycotoxins along the gut microbiota–liver axis, based on data compiled from Figures 1–4 and 44 publications (n=44). The mycotoxins included are AFB1 (aflatoxin B1), BEA (beauvericin), DON (deoxynivalenol), EENs (enniatins), FUM (fumonisin), OTA (ochratoxin A), and ZEN (zearalenone). This figure highlights how these toxins interact with or affect the gut–liver axis, providing a visual summary of their prevalence and impact across the studies analyzed.

**Table 4S.** Presentation of the frequency of studies according to the type of mycotoxin investigated along the gut microbiota–liver axis. The mycotoxins included are AFB1, OTA, DON, ZEN, FBs, ENNs, BEA, and T-2 toxin. This table highlights which mycotoxins have been most frequently studied across the analyzed publications, providing an overview of research focus in this area.

| **Mycotoxin** | **Number of studies** |
| --- | --- |
| AFB1 | 20 |
| OTA | 9 |
| DON | 9 |
| ZEN | 6 |
| FBs | 2 |
| ENNs | 1 |
| BEA | 1 |
| T-2 | 1 |

AFB1: Aflatoxin B1; BEA: Beauvericin; DON: Deoxynivalenol; ENNs: Enniatins; FBs: Fumonisins; OTA: Ochratoxin A; ZEN: Zearalenone.

**Figure 11S.** Distribution of animal species studied in relation to the gut microbiota–liver axis, based on data collected from Tables 1–4 and 44 publications (n=44). The figure includes broilers, mice, piglets, fish, sheep, hens, cows, ducklings, and rabbits, highlighting which animal models are most commonly used to investigate interactions between the gut microbiota and liver function.

**Table 5S.** Summary of the frequency of studies conducted for each animal species in relation to the gut microbiota–liver axis. The table includes data for broilers, mice, piglets, fish, sheep, hens, cows, ducklings, and rabbits, showing how often each species has been used in the 44 publications analyzed. This provides an overview of the most commonly studied animal models in this field.

| **Animal species** | **Number of studies** |
| --- | --- |
| Broilers | 10 |
| Mice | 15 |
| Piglets | 6 |
| Fish | 3 |
| Sheep | 3 |
| Hens | 1 |
| Cows | 1 |
| Ducklings | 4 |
| Rabbits | 1 |


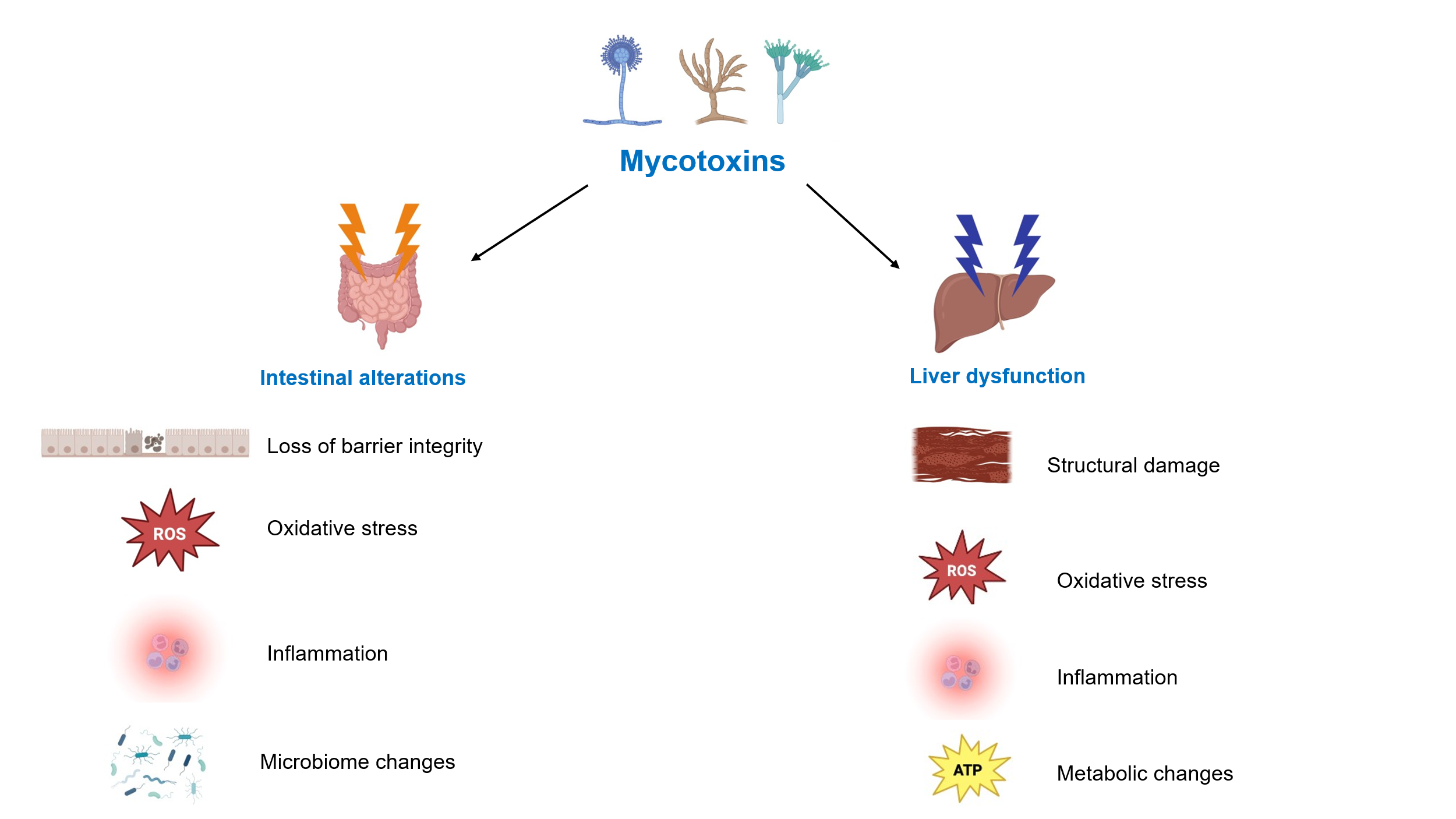


**Figure 12S.** Overview of mycotoxins examined in relation to intestinal alterations and liver dysfunction, highlighting intestinal barrier disruption, oxidative stress, inflammation, microbiome alterations, hepatic structural damage, and metabolic impairment.

**Supplementary comment:**

Recent updates from the Genome Taxonomy Database (GTDB, release R10‑RS226, 2025) have revised the classification of many prokaryotic lineages. Traditional phylum names such as Firmicutes and Proteobacteria are now formally recognized as Bacillota and Pseudomonadota, respectively. Similarly, Bacteroidetes has been updated to Bacteroidota (Parks et al., 2025). Other relevant phyla include Patescibacteriota, Campylobacterota, Verrucomicrobiota, and Cyanobacteriota, reflecting a genome-based phylogenomic framework. These updates improve consistency and reproducibility in microbiome research. GTDB also highlights that a significant fraction of microbial diversity remains uncharacterized, with many species yet to be genomically resolved (Parks et al., 2025). Additionally, more than 20,000 “Candidatus” taxa have been assigned formal Latin names, standardizing previously informal lineages. Incorporating these updated classifications ensures accurate reporting of gut microbiota composition. This is particularly important when interpreting shifts in microbial populations associated with interventions such as mycotoxin exposure. Overall, using GTDB taxonomy enhances the rigor and comparability of microbiome studies (Parks et al., 2025).
